# *Globularia alypum* L. and Related Species: LC-MS Profiles and Antidiabetic, Antioxidant, Anti-Inflammatory, Antibacterial and Anticancer Potential

**DOI:** 10.3390/ph15050506

**Published:** 2022-04-21

**Authors:** Maja Friščić, Roberta Petlevski, Ivan Kosalec, Josip Madunić, Maja Matulić, Franz Bucar, Kroata Hazler Pilepić, Željan Maleš

**Affiliations:** 1 Department of Pharmaceutical Botany, Faculty of Pharmacy and Biochemistry, University of Zagreb, Schrottova 39, HR-10000 Zagreb, Croatia; zeljan.males@pharma.unizg.hr; 2 Department of Medical Biochemistry and Hematology, Faculty of Pharmacy and Biochemistry, University of Zagreb, Domagojeva 2, HR-10000 Zagreb, Croatia; roberta.petlevski@pharma.unizg.hr; 3 Department of Microbiology, Faculty of Pharmacy and Biochemistry, University of Zagreb, Schrottova 39, HR-10000 Zagreb, Croatia; ivan.kosalec@pharma.unizg.hr; 4 Biochemistry and Organic Analytical Chemistry Unit, Institute for Medical Research and Occupational Health, Ksaverska cesta 2, HR-10001 Zagreb, Croatia; jmadunic@imi.hr; 5 Department of Biology, Division of Molecular Biology, Faculty of Science, University of Zagreb, Horvatovac 102A, HR-10000 Zagreb, Croatia; maja.matulic@biol.pmf.hr; 6 Department of Pharmacognosy, Institute of Pharmaceutical Sciences, University of Graz, Beethovenstraße 8, A-8010 Graz, Austria; franz.bucar@uni-graz.at

**Keywords:** *Globularia alypum*, metabolite profiling, hyperglycemia-induced oxidative stress, antiradical activity, cyclooxygenase-1, well diffusion, broth microdilution, MRSA, MDA-MB-231, A1235

## Abstract

Species from the genus *Globularia* L. have been used as healing agents for various ailments, with utilization of *Globularia alypum* L. being most frequently reported. The aim of this study was to evaluate the antidiabetic, antioxidant, anti-inflammatory, antibacterial and anticancer potential of *G. alypum* and three related species, *G. punctata* Lapeyr., *G. cordifolia* L. and *G. meridionalis* (Podp.) O.Schwarz, in relation to their phytochemical compositions. Globularin and verbascoside were identified using LC-PDA-ESI-MS^n^ as the major metabolites of *G. alypum* with known biological activities. *G. alypum* demonstrated the greatest α-glucosidase inhibitory activity and DPPH radical scavenging activity (IC_50_ = 17.25 μg/mL), while its anti-inflammatory activity was not significantly different from those of related species. All investigated species showed considerable antibacterial activity against methicillin-resistant *Staphylococcus aureus* in the broth microdilution method (MIC = 1.42–3.79 mg/mL). *G. punctata* also showed antibacterial activities against *Escherichia coli* (MIC = 1.42 mg/mL), *Bacillus subtilis* (MIC = 1.89 mg/mL), *B. cereus* (MIC = 2.84 mg/mL) and *Enterococcus faecalis* (MBC = 5.68 mg/mL). *G. punctata*, *G. cordifolia* and *G. meridionalis* showed greater anticancer potential than *G. alypum*. Obtained results indicate investigated *Globularia* species could serve as sources of diverse bioactive molecules, with *G. punctata* having the greatest antibacterial potential.

## 1. Introduction

According to the World Health Organization (WHO) statistics for 2021, the leading causes of global premature mortality from non-communicable diseases include cancer, cardiovascular diseases, diabetes, and chronic respiratory diseases. In 2019, 33.2 million people died solely from these diseases, which is 28% more deaths caused by the same four diseases than in 2000. Taken individually, there has been a 25% increase in the total global mortality from cardiovascular diseases (17.9 million deaths), a 37% increase in cancer mortality (9.3 million deaths), and a 10% increase in chronic respiratory diseases mortality (4.1 million deaths), while diabetes mortality has grown by 72% [1]. According to the 2019 estimates obtained from the International Diabetes Federation, about 4.2 million deaths among people aged 20 to 79 years may be attributed to diabetes [2].

Chronic diseases, including cardiovascular diseases (CVD), diabetes, cancer, and neurodegenerative diseases, are closely related to oxidative stress and inflammation [3,4]. Development and progression of diabetes as well as the occurrence of diabetes-associated macro- (CVD) and microvascular complications, such as neuropathy, retinopathy, nephropathy, and diabetic foot, are attributable to hyperglycemia-induced excessive reactive oxygen species (ROS) production and reduced antioxidant response [5,6]. Insulin resistance and pancreatic β-cell dysfunction that lie in the background of hyperglycemia are closely correlated with mitochondrial dysfunction, endoplasmic reticulum stress and inflammation [5]. Foot ulcers that occur in up to 25% of diabetic patients may often progress to diabetic foot infections, which are the single most important cause of diabetes-associated hospitalizations. These infections are often associated with a 5-year mortality rate of about 40% and are the major reason for nontraumatic lower extremity amputations [7].

Plants, thanks to the presence of numerous secondary metabolites and their diverse biological activities, help alleviate various ailments and/or may serve as sources of lead compounds in the discovery of new drugs [8]. According to biological activity studies, *Globularia alypum* L. is one of the plants that may help alleviate symptoms of different chronic diseases [9,10,11,12]. Its medicinal use is especially well-documented in North African countries, such as Morocco and Algeria, where it is one of the most frequently cited plant species used for diabetes [13,14]. It was also recorded as one of the plants used with high fidelity level for wounds and/or burns by both herbalists and housewives [15]. Treatment of foot ulcers often developed by diabetic patients has also been documented [14]. A study conducted from 2014 to 2017 in the Eastern Region of Libya found that, out of 179 plant species mentioned by informants, *G. alypum* had the maximum recorded treatment value of 10.9%. It was used as a diuretic agent, hemostatic, ovary stimulant and abortive, as well as for gastritis, hypertension, metritis, stroke, vaginal diseases, diarrhea, ulcer, colic, eczema, psoriasis, dermatitis, vaginitis, premenstrual syndrome, and delayed menses [16].

While records of antidiabetic and anticancer utilization are more often indicating the use of *G. alypum* (and its close relative *G. arabica* Jaub. and Spach) leaves subjected to infusion or decoction [13,17,18], aerial parts (flowering tops) in the form of powder or decoction seem to be chosen more frequently for wound healing, acne, eczema, abscesses, and skin infections [19,20]. Antioxidant [11,12], anti-obesity, anti-hyperglycemic, anti-hyperlipidemic, anti-α-amylase, anti-lipase, anti-liver toxicity, anti-pyretic, analgesic [11], anti-inflammatory, anti-microbial and wound healing effects have been reported for *G. alypum* methanolic leaf extracts [12], while aqueous leaf extract of the same species showed in vitro cytotoxic effect against Hep*-*2 human laryngeal carcinoma cells and Vero cells [21]. Observed biological effects have mainly been attributed to its secondary metabolites, such as iridoids and/or polyphenols [10,11].

Similarities and differences in phytochemical composition between *G. alypum* and three related species, *G. punctata* Lapeyr., *G. cordifolia* L. and *G. meridionalis* (Podp.) O.Schwarz, were already highlighted in our previous paper based on the liquid chromatography-photodiode array detection-electrospray ionization-tandem mass spectrometry (LC-PDA-ESI-MS^n^) analysis of phenolic and iridoid constituents from methanolic extracts of aerial parts obtained by boiling under reflux conditions [22]. We have also observed that the same three species possessed comparable or even greater amounts of polyphenolic compounds, flavonoids, tannins and/or iridoids in their aerial parts than *G. alypum* [23,24], while observed antioxidant activity of *Globularia* extracts [23] goes in favor of their application in cosmetics and the food industry. Extracts obtained from plant cell cultures of *G. cordifolia* have already found their commercial use in cosmetic products [25]. *G. cordifolia* is also a natural source of the iridoid glycoside globularifolin, which has shown free radical scavenging, anti-inflammatory, and immunomodulatory potential [26], as well as anticancer activity against various cancer cell lines, including U87 human glioblastoma cells and CAMA-1 human breast cancer cells [27,28]. On the other hand, antioxidant, anti-acetylcholinesterase and anti-butyrylcholinesterase activities were reported for *G. meridionalis* [29], which has comparable phytochemical composition to *G. cordifolia* [22,23]. The same activities, together with α-glucosidase and α-amylase, tyrosinase and lipase inhibitory activity, were recently reported for *G. orientalis* L. and *G. trichosantha* Fisch. and C.A.Mey. [30], of which the latter belongs to the same section as *G. punctata* [31], the most widely distributed *Globularia* species in Europe [32].

In order to improve the understanding of the multiple ethnobotanical uses of species from the genus *Globularia* reported in the literature, most frequently for *G. alypum*, and given the overall data scarcity considering the biological activities of species from the genus *Globularia* other than *G. alypum*, the aims of the present study were the following: (1) to evaluate the phytochemical composition of *G. alypum*, *G. punctata*, *G. cordifolia* and *G. meridionalis* methanolic leaf extracts obtained by ultrasound-assisted extraction; (2) to evaluate the phytochemical composition of *G. alypum*, *G. punctata*, *G. cordifolia* and *G. meridionalis* methanolic aerial parts extracts obtained by Soxhlet extraction; (3) to evaluate the antidiabetic, antioxidant and anticancer potential of phytochemically characterized *Globularia* leaf extracts; (4) to evaluate the antioxidant, anti-inflammatory and antimicrobial potential of phytochemically characterized *Globularia* aerial parts extracts; and (5) to consider constituents of investigated *Globularia* spp. potentially responsible for the observed biological effects.

Compared to *G. alypum*, species *G. punctata*, *G. cordifolia* and *G. meridionalis* have a relatively great distribution in Europe [32]. The two different methods (ultrasound-assisted extraction of leaves and Soxhlet extraction of aerial parts) used for the preparation of *Globularia* extracts were chosen, taking into consideration different plant parts and methods of preparation that were common according to recorded traditional applications of *G. alypum* and related species reported in ethnobotanical/ethnomedicinal literature from different countries (e.g., maceration and/or decoction). Prepared extracts were phytochemically characterized using UV/Vis spectrophotometry, LC-PDA-ESI-MS^n^ and thin-layer chromatography (TLC) and subjected to further biological activity testing. Antidiabetic (and antioxidant) potential has been evaluated based on the assessment of α-glucosidase activity and oxidative stress biomarkers, of which two were enzymatic (glutathione S-transferase (GST) and glutathione peroxidase (GPx)), and two were non-enzymatic (free thiol groups (-SH) and reduced glutathione (GSH)), in Hep G2 cells cultured under hyperglycemic conditions. Further, cell viability was measured by applying two commonly used assays, lactate dehydrogenase (LDH) and 3-(4,5-dimethylthiazol-2-yl)-2,5-diphenyltetrazolium bromide (MTT) assay. Additionally, antiradical activity against the DPPH free radical was assessed, both spectrophotometrically and by TLC bioautography. Anti-inflammatory potential was evaluated by measuring the inhibitory effect on cyclooxygenase-1 (COX-1) activity using two different methods (*N*,*N*,*N′*,*N′*-tetramethyl-*p*-phenylenediamine dihydrochloride (TMPD assay) and the prostaglandin E_2_ assay (PGE_2_ assay)). Antibacterial potential against four Gram-positive and three Gram-negative bacterial species, including the pathogens that are commonly found in diabetic foot infections, such as *Staphylococcus aureus* (*S. aureus*) and methicillin-resistant *S. aureus* (MRSA) [7], was evaluated using well-diffusion and/or serial broth microdilution, followed by agar sub-cultivation, as complementary methods. Finally, anticancer potential was assessed by measuring cell viability of two different human cancer cell lines, MDA-MB-231 breast cancer cell line and A1235 glioblastoma cell line (MTT assay).

The obtained results confirm previously reported biological activities of *G. alypum* and provide a better understanding of its well-known traditional uses, while also offering some new considerations that have not been discussed in previous papers. For example, the observed anti-staphylococcal activity in combination with anti-inflammatory and antioxidant activity goes in favor of the recorded external use of *G. alypum* for different skin disorders, including healing of diabetic foot ulcers, wounds, abscesses, and burns. To date, the antidiabetic potential of *G. alypum* has mainly been evaluated from the perspective of its oral administration. The results of our study also give a better insight into the medicinal potential of the related, well-distributed European species, while pointing out the metabolites of possible interest for further bioactivity studies. To our knowledge, this is the first study comparing the antidiabetic, anti-inflammatory, antimicrobial, and anticancer potential of several *Globularia* species, including the most well investigated *G. alypum* and, to date, the most extensive study of biological activities of the species from the genus *Globularia* accompanied with detailed evaluation of their phytochemical compositions.

## 2. Results and Discussion

### 2.1. Phytochemical Content

#### 2.1.1. Phytochemical Content of Leaf Extracts

Contents of secondary metabolites in leaf extracts used for evaluation of antidiabetic and anticancer potential are summarized in Table 1. In leaf extracts used for evaluation of antidiabetic activity, the highest amounts of polyphenols were observed for *G. alypum* (130.46 mg GAE/g DE) and *G. meridionalis* (123.44 mg GAE/g DE) (*p* < 0.05). On the other hand, total phenolic contents of leaf extracts of *G. punctata*, *G. cordifolia* and *G. meridionalis* used for anticancer potential evaluation were higher than that of *G. alypum* (*p* < 0.05).

Consistent with our previous findings [23], levels of all other secondary metabolites were significantly higher in *G. punctata*, *G. cordifolia* and *G. meridionalis* when compared to those of the medicinal plant *G. alypum* (*p* < 0.05). Thereby, *G. punctata* contained the highest amounts of flavonoids (48.49–63.03 mg QE/g DE) and iridoids (343.33–440.04 mg AE/g DE) (*p* < 0.05), while *G. cordifolia* and *G. meridionalis* were characterized by higher condensed tannin contents (6.21–10.02 mg CE/g DE).

As previously mentioned [23], the method used for the evaluation of iridoid content was less informative for *G. alypum* extracts due to its inability to detect catalpol-type iridoids, which seem to be dominant in this species [22], while it could detect asperuloside-type iridoids [23], characteristic for the remaining three species [22]. On the other hand, presence of condensed tannin monomers (e.g., (epi)gallocatechin, catechin) was rarely reported in *G. alypum* [33,34]. With this in mind, iridoid content and condensed tannin content were not subsequently evaluated in Soxhlet extracts of aerial parts.

#### 2.1.2. Phytochemical Content of Aerial Parts Extracts

In comparison to leaf extracts, lower values of secondary metabolites were observed in aerial parts extracts used for evaluation of antimicrobial, anti-inflammatory, and antioxidant activity (Table 2). This agrees with our previous report [23], according to which the stem extract of *G. alypum* and flower extracts of related species may contain less polyphenols than their leaf extracts. The aerial parts extract from *G. punctata* was characterized by lowest total phenolic content (79.92 mg GAE/g DE), while that of *G. alypum* contained the highest amount of phenolics (112.34 mg GAE/g DE). However, the *G. punctata* extract contained the highest flavonoid content (43.25 mg QE/g DE) (*p* < 0.05). Significant differences in total phenolic and flavonoid contents between *G. cordifolia* and *G. meridionalis* extracts were not observed (*p* > 0.05), which is also consistent with our previous findings [23].

### 2.2. LC-MS Profile

#### 2.2.1. Compound Identification and LC-MS Profile of Leaf Extracts

**Figure 1:**
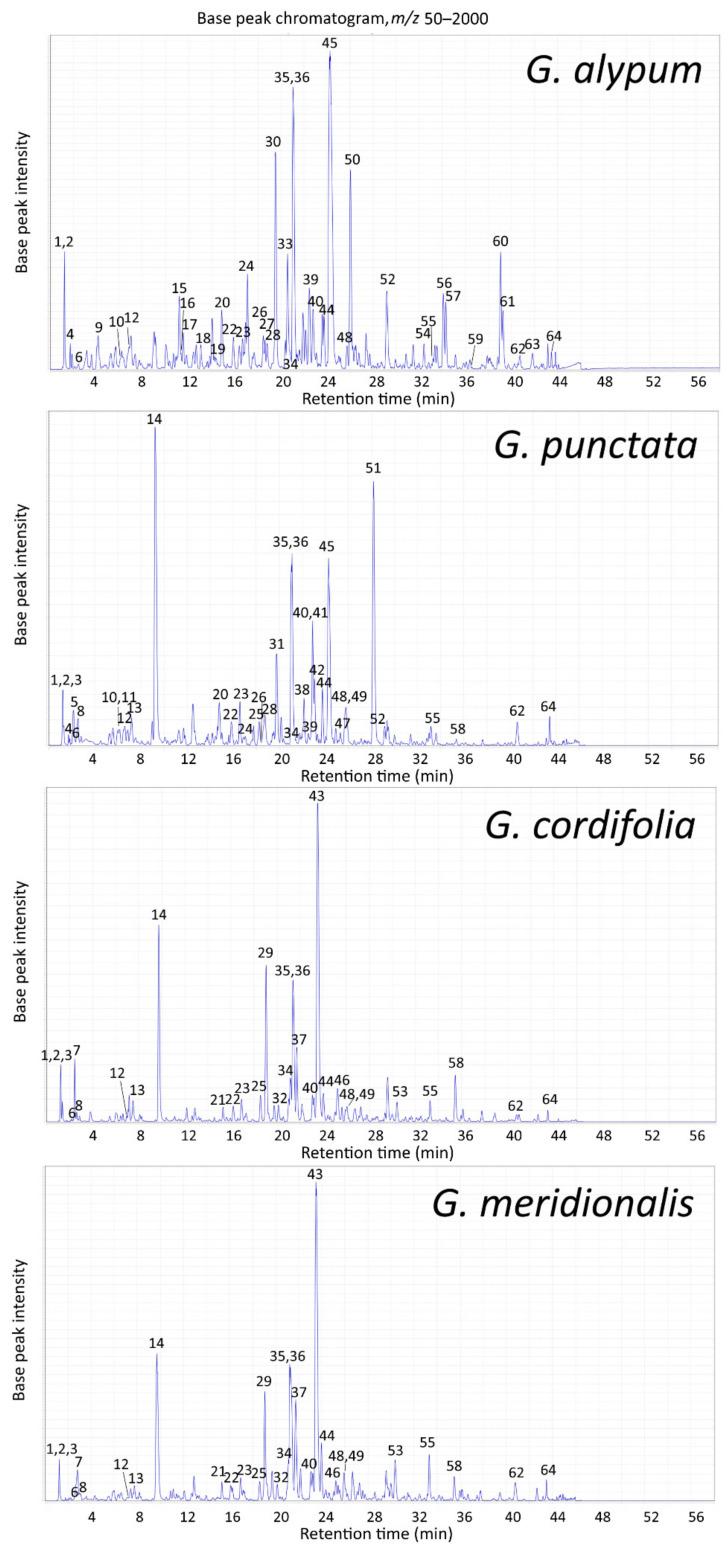
LC-MS base peak chromatograms of methanolic leaf extracts from *G. alypum*, *G. punctata*, *G. cordifolia* and *G. meridionalis* obtained by ultrasound-assisted extraction. Numbers on chromatograms refer to compounds listed in Table 3.

The liquid chromatography-photodiode array detection-electrospray ionization-tandem mass spectrometry (LC-PDA-ESI-MS^n^) method previously applied for the separation and identification of methanolic extract constituents obtained by extraction under reflux conditions of *G. alypum*, *G. punctata*, *G. cordifolia* and *G. meridionalis* aerial parts [22] facilitated identification of newly observed constituents of their leaf extracts (Figure 1, Table 3). New constituents were identified based on the comparison of their retention time, UV and MS spectra, and MS/MS fragmentation pattern (Table S1) to published chromatographic and spectral data of recorded constituents [22] and other literature data considering metabolites reported for investigated *Globularia* species.

Compounds **9** (*m/z* 315), **50** (*m/z* 491) **56** (*m/z* 639), **61** (*m/z* 653) and **63** (*m/z* 789) were recognized as phenylethanoids characteristic for *G. alypum*. Compound **9** was tentatively identified as 1′-*O*-hydroxytyrosol glucoside (2-(3′,4′-dihydroxyphenyl)ethyl-*O*-β-d-glucopyranoside), a structural part of all identified phenylethanoids, based on the MS^2^ fragment ions present at *m/z* 135 [M−H−glucose]^−^ and 153 [M−H−anhydroglucose]^−^. In MS^2^, compound **50** was characterized by product ions at *m/z* 161 (−330 Da) and 175 (−316 Da). In MS^3^ of the first product ion, further loss of 28 Da (−CO) was observed. The first loss in MS^2^ was attributed to simultaneous loss of hydroxytyrosol glucoside and a methyl group and the second to hydroxytyrosol glucoside. Loss of a methyl group was previously observed for the reference standard of ferulic acid [22]. The compound was tentatively identified as 6′-*O*-feruloyl-1′-*O*-hydroxytyrosol glucoside (= desrhamnosyl decaffeoylgalypumoside B = desrhamnosyl leucosceptoside A = methylated calceolarioside B) in comparison to other compounds detected in *G. alypum*, such as 6′-*O*-(*p*-coumaroyl)-1′-*O*-hydroxytyrosol glucoside (neosyringalide), leucosceptoside A, galypumoside B, and globularitol (6′-*O*-feruloyl-β-d-glucopyranosyl-(1→6)-mannitol) [22,33,35,36]. Presence of dihydroxyphenylethyl-methylcaffeoyl-hexoside was recently reported for methanolic leaf extract of *G. alypum* [37]. Compounds **56** and **61** were characterized by UV spectra comparable to those of caffeoyl phenylethanoids, while their retention times matched those of diacylated phenylethanoids 6′-*O*-caffeoylverbascoside (**57**) and galypumoside B (**60**). In MS^2^ of compound **56**, loss of 162 Da was attributed to anhydrocaffeic acid loss, while those observed for compound **61** were attributed to anhydroferulic (−176 Da), ferulic (−194 Da) and anhydrocaffeic acid (−162 Da). The compounds were tentatively identified as desrhamnosyl 6′-*O*-caffeoylverbascoside and desrhamnosyl galypumoside B (= 4′-*O*-caffeoyl-6′-*O*-feruloyl-1′-*O*-hydroxytyrosyl glucoside). Phenylethanoids that contain two acyl groups and/or do not contain rhamnose (e.g., calceolarioside A and B) were observed only for this species.

Compound **63** was characterized by fragment ions present at *m/z* 461 (−328 Da) and 623 (−166 Da) with a dominant fragment present at *m/z* 627 (−162 Da) in MS^2^. From the latter, a loss of 166 Da was recorded in MS^3^, while in MS^4^ the obtained product ion (*m/z* 461) gave a fragmentation pattern characteristic for phenylethanoids, with the major fragment present at *m/z* 315 [hydroxytyrosol glucoside−H]^−^. The compound was tentatively identified as galypumoside C (6′-*O*-menthiafoloylverbascoside), whose presence in leaves of *G. alypum* was already reported [36,37]. Geniposide (**17**), whose identity was confirmed based on comparison of chromatographic and spectral data to those previously observed for its reference standard [22], was another compound characteristic for *G. alypum*.

Compound **34** (*m/z* 477) was recorded in all four species but was more abundant in *G. cordifolia* and *G. meridionalis* leaf extracts. It was characterized by loss of 162 Da in MS^2^ [M–H−anhydroglucose]^−^. The formed radical aglycone product ion was further subjected to loss of one methyl group (−15 Da) in MS^3^ and major loss of 84 Da in MS^4^, attributed to the cleavage of three CO [22]. The compound was identified as nepetin 7-*O*-glucoside (6-methoxyluteolin-7-*O*-glucoside), a flavone previously isolated from *G. dumulosa* [38], whose presence was also recently reported in *G. alypum* leaf extract [37].

Other constituents tentatively identified in *G. cordifolia* and *G. meridionalis* were phenylethanoids **37** (*m/z* 653) and **58** (*m/z* 889) (detected also in *G. punctata*) and iridoids **21** (*m/z* 701) and **25** (*m/z* 493) (detected also in *G. punctata*). MS^2^ of compound **37** indicated a loss of 192 Da, while the following MS^3^ and MS^4^ spectra matched those of verbascoside/isoverbascoside. Considering other phenylethanoids that were observed for these two species [22,29,39], which were either caffeic (verbascoside, isoverbascoside) or ferulic acid derivatives (leucosceptoside A, plantainoside C, martynoside), the observed loss of 192 Da was attributed to the presence of 3-methoxycaffeic acid and the compound was tentatively identified as methoxyverbascoside isomer. Second-order MS of compound **58** indicated major loss of 122 Da (*m/z* 767), while further MS/MS fragmentation pattern matched that of rossicaside A isomer. Additional fragments present at *m/z* 605 (−284 Da) and 727 (−162 Da) indicated loss of anhydrocaffeic acid with or without benzoic acid and the compound was tentatively identified as benzoylrossicaside A isomer.

The MS^2^ of compound **21** indicated dominant loss of anhydroglucose with formic acid (−208 Da) or loss of formic acid (−46 Da). Presence of benzoic acid was indicated by UV λ_max_ (195 and 238 nm) and observed cleavage of the major MS^2^ product ion (*m/z* 493), attributed to simultaneous loss of benzoylanhydroglucose (−266 Da) and CO_2_ (−44 Da). This, together with minor fragments present at *m/z* 165 [M–H–anhydroglucose–benzoylglucose−CO_2_]^−^, 209 [M–H–anhydroglucose−benzoylglucose]^−^, 227 [M–H–anhydroglucose−benzoylanhydroglucose]^−^ and 371 [M–H–anhydroglucose−benzoic acid]^−^, indicated a similar structure to previously identified C-4 carboxylated iridoids. Second- and third-order mass spectra of compound **25** were comparable to MS^3^ and MS^4^ of compound **21**, while major loss of 30 Da (−CH_2_O) from the MS^3^ product ion present at *m/z* 165 was observed in MS^4^ together with minor fragment ions present at *m/z* 121 (−C_2_H_4_O), 137 (−CO) and 147 (−H_2_O). These compounds were tentatively identified as 6′-*O*-benzoyldeacetylasperulosidic acid glucoside and 6′-*O*-benzoyldeacetylasperulosidic acid, keeping in mind other observed asperuloside-type iridoids and benzoylation of glucose at C-6′ position, which was recorded for dumuloside in *G. dumulosa* [38] and aphyllanthoside in *G. punctata* [40].

Compound **49** (*m/z* 517), which also showed a minor formic acid adduct in MS^1^, characteristic for many iridoids, was only found in *G. cordifolia*, *G. meridionalis* and *G. punctata*. Dominant loss of 326 Da in MS^2^ was attributed to anhydroglucose and *p*-coumaric acid. Minor fragments were also present at *m/z* 147 [M–H–anhydroglucose–*p*-coumaric acid–CO_2_]^–^, 163 [*p*-coumaric acid–H]^–^, 293 [M–H–glucose–CO_2_]^–^ and 355 [M–H–anhydroglucose]^–^. Further MS/MS fragmentation pattern obtained in MS^3^ and MS^4^ were in accordance with those of asperuloside and besperuloside, which have acetic/benzoic acid attached at C-10 position. This compound, with UV λ_max_ present at 191, 232 and 315 nm, comparable to those previously observed for asperuloside (191 and 239 nm) and *p*-coumaric acid (210, 227 and 311 nm) [22], was identified as 10-*O*-(*p*-coumaroyl)-deacetylasperuloside.

Obtained chromatogram of *G. alypum* methanolic leaf extract indicated not only the previously described high globularin (10-*O-trans*-cinnamoylcatalpol) content [41], but also high relative amounts of verbascoside, rossicaside A and other phenylethanoids. In addition to globularin, other catalpol-type iridoids, which, as previously mentioned [23], do not provide a reaction with the Trim–Hill reagent used for iridoid quantification, were also observed together with C-4 carboxylated iridoids (e.g., geniposide, alpinoside). The major flavonoids observed for *G. alypum* were glycosides of 6-hydroxyluteolin, the same as for other investigated species, while vicenin-2 was observed only in *G. alypum*, as well as the lignan diglucoside liriodendrin. Leaf extract of *G. punctata* contained high relative amounts of iridoids asperuloside, besperuloside, globularin, deacetylasperuloside, asperulosidic acid, and scandoside, while verbascoside, its isomers (isoverbascoside, forsythoside A) and glycosylated derivatives (rossicaside A, trichosanthoside A and B, arenarioside) were observed as the major phenylethanoids. Solely *G. punctata* was characterized by acylated derivatives of 6-hydroxyluteolin, as previously reported [22,40,42]. *G. cordifolia* and *G. meridionalis* were characterized by iridoids asperuloside and monomelittoside and their often benzoylated derivatives, with globularifolin (10-*O*-benzoylmonomelittoside) as the major compound, as previously reported [22,26,43]. Major phenylethanoids observed for these species were verbascoside, rossicaside A and methoxyverbascoside isomer followed by isoverbascoside, forsythoside A, benzoylrossicaside A isomer and globusintenoside isomer. Nepetin 7-*O*-glucoside and demethoxycentaureidin 6,4′-dimethyl ether were recognized as characteristic flavonoids. Other major constituents observed in investigated *Globularia* species included mannitol, sucrose, quinic acid (all species except for *G. alypum*) and fatty acid oxidation products that have already been reported [22].

#### 2.2.2. LC-MS Profile of Aerial Parts Extracts

**Figure 2:**
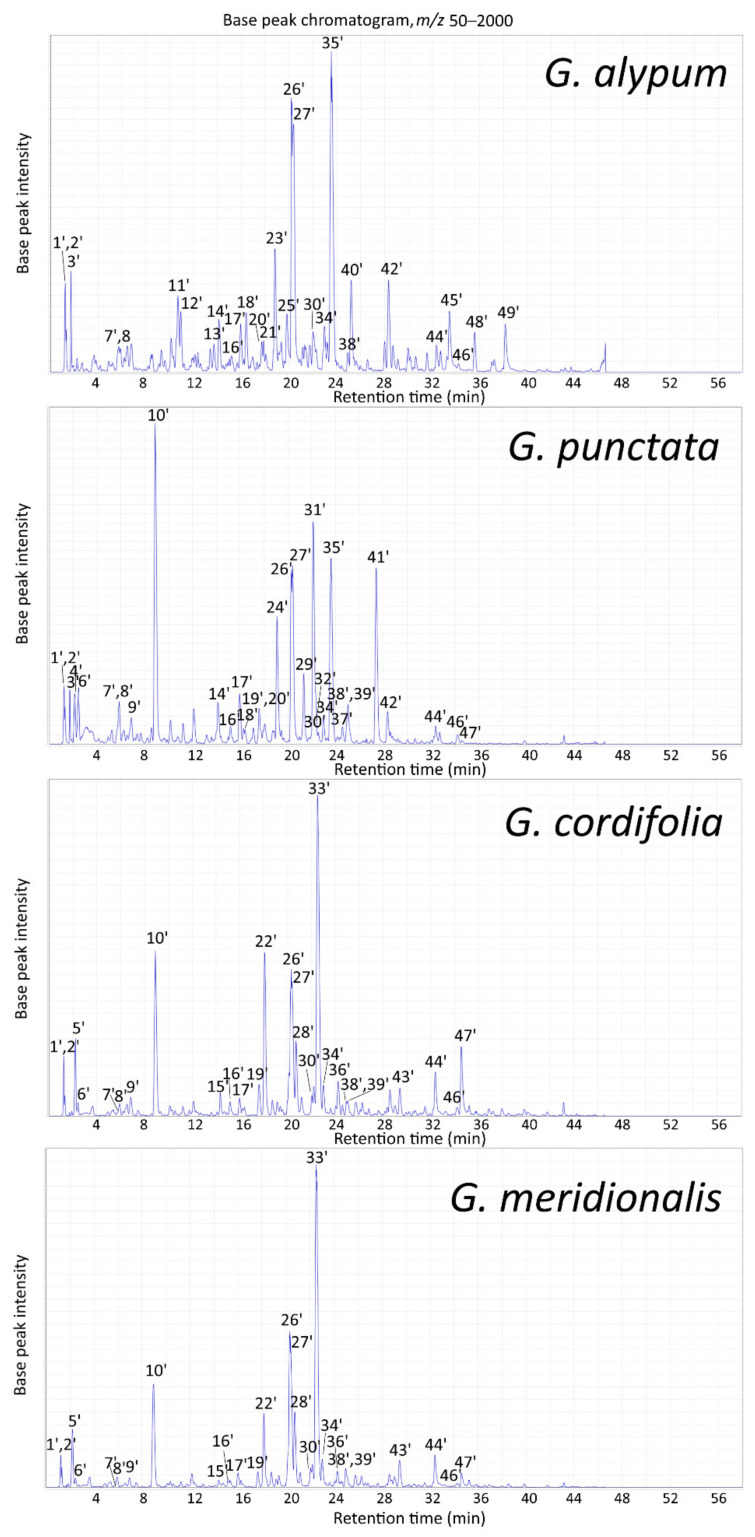
LC-MS base peak chromatograms of methanolic aerial parts extracts from *G. alypum*, *G. punctata*, *G. cordifolia* and *G. meridionalis* obtained by Soxhlet extraction. Numbers on chromatograms refer to compounds listed in Table 4.

Identification of major constituents of methanolic aerial parts extracts of investigated *Globularia* species obtained by Soxhlet extraction was performed using LC-PDA-ESI-MS^n^ (Figure 2, Table 4) in comparison to established compositions of leaf extracts (Figure 1, Table 3 and Table S1) or those of aerial parts extracts obtained by boiling under reflux conditions [22]. For *G. alypum*, these included mannitol, sucrose, catalpol, verminoside, geniposide, 6-hydroxyluteolin 7*-O-*sophoroside, alpinoside, globularinin, calceolarioside A, calceolarioside B, rossicaside A, verbascoside, isoverbascoside, forsythoside A, globularin, 6′-*O*-feruloyl-1′*-O-*hydroxytyrosol glucoside, globularioside, 6′-*O*-caffeoylverbascoside, globuloside A and galypumoside B. *G. punctata* was characterized by mannitol, sucrose, catalpol, 6-hydroxyluteolin 7-*O*-sophoroside, alpinoside, rossicaside A, verbascoside, isoverbascoside, globularin and globularioside, together with its distinctive compounds scandoside, 6-hydroxyluteolin 7*-O-*(6′′′-*O*-caffeoyl)-sophoroside, trichosanthoside B, trichosanthoside A, arenarioside, and besperuloside, with the iridoid asperuloside as the major compound. Other more prominent peaks included deacetylasperuloside and 10-*O*-(*p-*coumaroyl)-deacetylasperuloside, present also in *G. cordifolia* and *G. meridionalis*, as well as gardoside, caffeoylglucoside isomer and globusintenoside isomer present in all four species. For *G. cordifolia* and *G. meridionalis*, the major compounds were mannitol, sucrose, asperuloside and several phenylethanoids, including verbascoside, methoxyverbascoside isomer, isoverbascoside, globusintenoside isomer and benzoylrossicaside A isomer, while their distinctive peaks could be attributed to monomelittoside, 6′-*O*-benzoylmonomelittoside and globularifolin. The major identified flavonoid characteristic for these two species was demethoxycentaureidin 6,4′-dimethyl ether. Besides 6-hydroxyluteolin 7-*O*-glucoside, apigenin was detected in Soxhlet extracts of all investigated species.

The greatest differences in major constituents between LC-MS profiles of leaf and aerial parts extracts were observed for *G. alypum* (Figure 1 and Figure 2). Keeping in mind the shrubby nature of the plant, this could partially be explained by a relatively higher share of stems in *G. alypum* aerial parts subjected to extraction than in related species. Stems of *G. alypum* were recorded to be less rich in secondary metabolites than its leaves and flowers [23,44,45]. Most of the major constituents of Soxhlet extracts were present with higher relative abundances than in the extracts prepared by boiling under reflux conditions [22].

#### 2.2.3. TLC Profile of Aerial Parts Extracts

**Figure 3:**
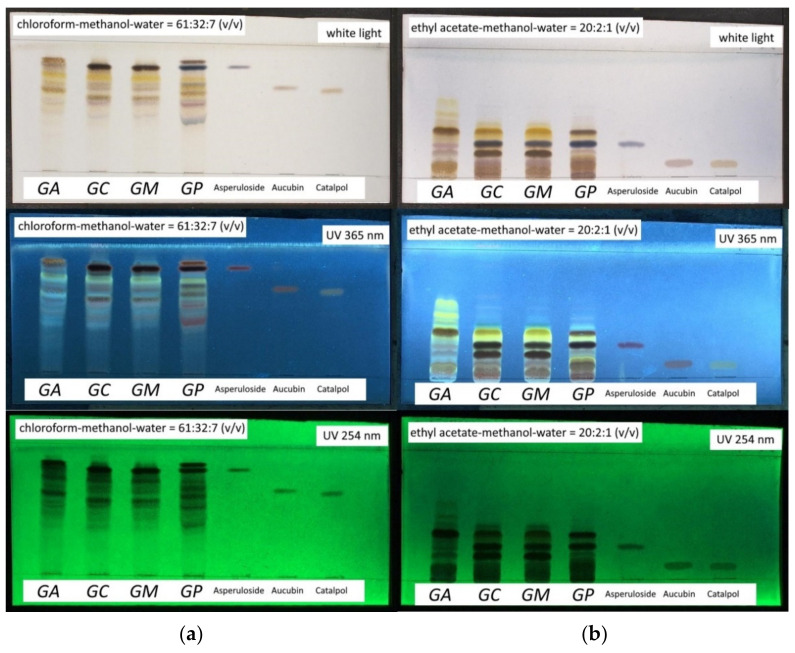
TLC chromatograms of Soxhlet extracts of aerial parts from *G. alypum* (*GA*), *G. cordifolia* (*GC*), *G. meridionalis* (*GM*) and *G. punctata* (*GP*) and asperuloside, aucubin and catalpol reference standard obtained after treatment with 1% (*w/v*) ethanolic vanillin-5% (*v/v*) ethanolic sulfuric acid reagent (5–10 min at 100–105 °C), observed under white light, UV 365 nm and UV 254 nm. Stationary phase: silica gel 60 F_254_; mobile phase: (**a**) chloroform-methanol-wate*r =* 61:32:7 (*v/v/v*); (**b**) ethyl acetate-methanol-wate*r =* 20:2:1 (*v/v/v*).

In addition to the performed LC-MS profiling, characteristic iridoid and phenylethanoid constituents of obtained Soxhlet extracts of aerial parts from *G. alypum*, *G. punctata*, *G. cordifolia* and *G. meridionalis* were also recorded by thin-layer chromatography (TLC) using two different mobile phases (Figure 3), as described previously [36]. This enabled faster and simpler observation of unique chemical fingerprints of *G. alypum*, *G. punctata* and *G. cordifolia*/*G. meridionalis*. Based on the comparison to standard compounds and/or to respective LC-MS chromatograms of investigated species (Figure 2, Table 4), two prominent brown zones observed under white light after treatment with vanillin-sulfuric acid reagent in *G. alypum* and *G. punctata* extract were attributed to globularin and catalpol, and those observed for *G. cordifolia* and *G. meridionalis* extract to globularifolin and monomelittoside and/or its 6′*-O-*benzoylated derivative as one of their major metabolites. Asperuloside, present in *G. punctata*, *G. cordifolia* and *G. meridionalis*, gave a blue zone, while two additional blue zones, which were more prominent in *G. punctata*, may be attributed to other identified asperuloside-type iridoids (e.g., asperulosidic acid and deacetylasperuloside). Blue zone of besperuloside, present only in *G. punctata*, was not clearly visible due to its overlapping with the brown zone of globularin. Shared yellow zones may be attributed to phenylethanoids present in all investigated species with verbascoside as the major shared component, while additional yellow zones observed for *G. alypum* and *G. punctata* correspond to the presence of their major unique phenylethanoids (e.g., for the latter, trichosanthoside A and B) that were detected using LC-MS (Table 4).

### 2.3. Antidiabetic and Antioxidant Potential

Antidiabetic potential of leaf extracts of studied species was evaluated based on their α-glucosidase inhibitory activity and effect on two enzymatic (glutathione S-transferase (GST) and glutathione peroxidase (GPx)) and two non-enzymatic biomarkers of oxidative stress (free thiol groups (-SH) and reduced glutathione (GSH)) in human hepatocellular carcinoma Hep G2 cells exposed to hyperglycemic conditions. Cell viability was evaluated using lactate dehydrogenase (LDH) and 3-(4,5-dimethylthiazol-2-yl)-2,5-diphenyltetrazolium bromide (MTT) assay. Antioxidant potential of aerial parts extracts was evaluated based on spectrophotometric measurement of 2,2-diphenyl-1-picrylhydrazyl (DPPH) radical scavenging activity and by performing thin layer chromatography (TLC) bioautography using the same free radical as spray reagent.

#### 2.3.1. α-Glucosidase Inhibition

**Figure 4:**
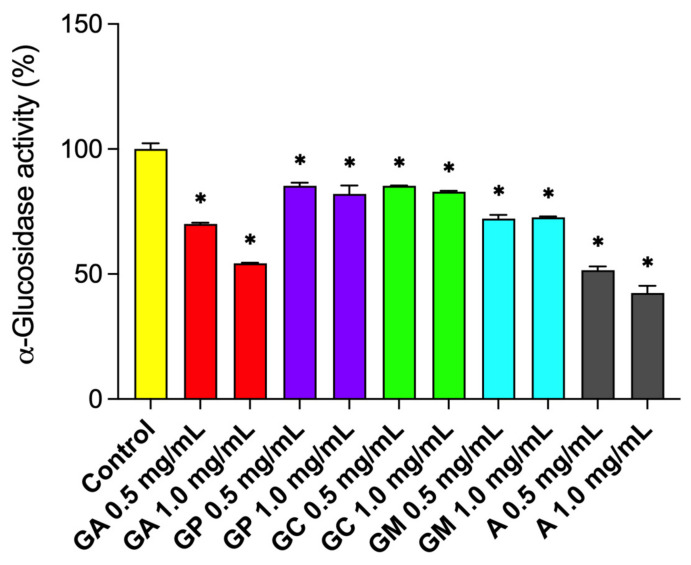
Effect of *G. alypum* (*GA*), *G. punctata* (*GP*), *G. cordifolia* (*GC*) and *G. meridionalis* (*GM*) methanolic leaf extracts on *α*-glucosidase activity (%); A—acarbose (positive control); * statistically significant difference in comparison to control (*p* < 0.05); values are means + SD (*n* = 4).

Control of the blood glucose level is the main strategy for treating diabetes mellitus (DM) and reducing diabetic complications. This can partially be achieved by inhibiting the activity of α-glucosidase, the key enzyme responsible for hydrolytic cleavage of complex carbohydrates. Inhibition of α-glucosidase may retard the absorption of glucose and decrease postprandial blood glucose levels and, therefore, it is one of the key targets for treating type 2 diabetes mellitus (T2DM) [46]. All four species caused significant inhibition of α-glucosidase activity at the concentrations tested (0.5 and 1.0 mg/mL) (*p* < 0.05), with *G. alypum* extracts being most effective (30.0–45.7% inhibition) (Figure 4). This supports the frequently reported traditional use of *G. alypum* as an antidiabetic agent.

Inhibition of α-glucosidase (IC_50_ = 0.52 mg/mL) and α-amylase (IC_50_ = 0.57 mg/mL) was reported for diethyl ether fraction of *G. alypum* aerial parts extract [47]. The major chromatographic peaks observed at 280 nm that were left unidentified could most likely be attributed to verbascoside and globularin (based on their UV spectra) [22], which were observed as one of the main components of *G. alypum* in the present and in related studies [35,36,37,48].

Moderate, but statistically non-significant negative correlation, was observed between α-glucosidase activity and total phenolic content (*r =* −0.51, *p* > 0.05) (Table S2). Inhibition of α-glucosidase, with very good negative correlation to total phenolic content (*r =* −0.791), was previously reported for *G. trichosantha* and *G. orientalis* [30]. In a study conducted on isolated compounds of *Clerodendrum bungei* Stud., the phenylethanoids verbascoside (IC_50_ = 0.5 mM), leucosceptoside A (IC_50_ = 0.7 mM) and isoverbascoside (IC_50_ = 0.1 mM), which were found in all investigated *Globularia* species, exhibited stronger anti-α-glucosidase activities than the positive control (acarbose) [49]. The observed effect might be due to the presence of the hydroxytyrosyl moiety (IC_50_ = 0.15 mM) [50] and/or trans-cinnamic acid derivatives (e.g., caffeic and ferulic acid) [51] in their structure. The named acids were found in the structure of most phenylethanoids of investigated *Globularia* species. Hereby, many acylated phenylethanoids were only found in *G. alypum*, including galypumoside B, calceolarioside A, and calceolarioside B, of which the latter was recently identified also as a pan inhibitor of SARS-CoV-2 proteins [52]. Phenolic acids were also present in iridoids characteristic for *G. alypum*, such as verminoside (caffeic acid) and specioside (*p-*coumaric acid). However, greater inhibition was observed for leaf extracts of *G. alypum* and *G. meridionalis* (Figure 4), which contained more phenolics and less iridoids (Table 1).

The observed inhibitory effect of all four species may in part be connected to the presence of 6-hydroxyflavones (e.g., 6-hydroxyluteolin 7*-O-*glucoside). In *Origanum majorana* L. leaves, 6-hydroxyapigenin (scutellarein) (IC_50_ = 12 μM) and 6-hydroxyluteolin (IC_50_ = 10 μM) were recognized as the most potent inhibitors of α-glucosidase, while weaker inhibitions were observed for 6-hydroxyluteolin 7*-O-*glucoside (IC_50_ = 300 μM) and flavones lacking the 6-hydroxyl substituent, apigenin and luteolin (IC_50_ > 500 μM) [53]. The recorded greater inhibitory activity of *G. alypum* extract compared to those of extracts of related species may also be explained by presence of additional α-glucosidase inhibitors, such as vicenin-2 (IC_50_ = 270.53 μM) [54].

#### 2.3.2. Oxidative Stress Biomarkers

The liver plays a vital role in blood glucose level regulation both in physiological and pathological states. In DM, liver is among the primary organs susceptible to hyperglycemia-induced oxidative stress, which may result in irreversible oxidative modifications of its macromolecules that may lead to abnormal glycogen deposition, non-alcoholic fatty liver disease, fibrosis, cirrhosis, hepatocellular carcinoma, and other liver abnormalities [55]. Besides the observed anti-hyperglycemic effect of *G. alypum* [10,11,56], which can be partially attributed to its major metabolite globularin [41], additional benefits of traditional utilization of *G. alypum* as an antidiabetic could include prevention of diabetic complications through direct or indirect antioxidant activity, including radical scavenging activity [35,57], enhancement of antioxidant enzyme activity and/or elevation of levels of non-enzymatic antioxidants [56,58,59]. For example, in streptozotocin-induced diabetic rats, administration of *G. alypum* methanolic leaf extract reduced glycemia and glycosylated hemoglobin levels, and improved the redox status, especially in the liver [56].

**Figure 5:**
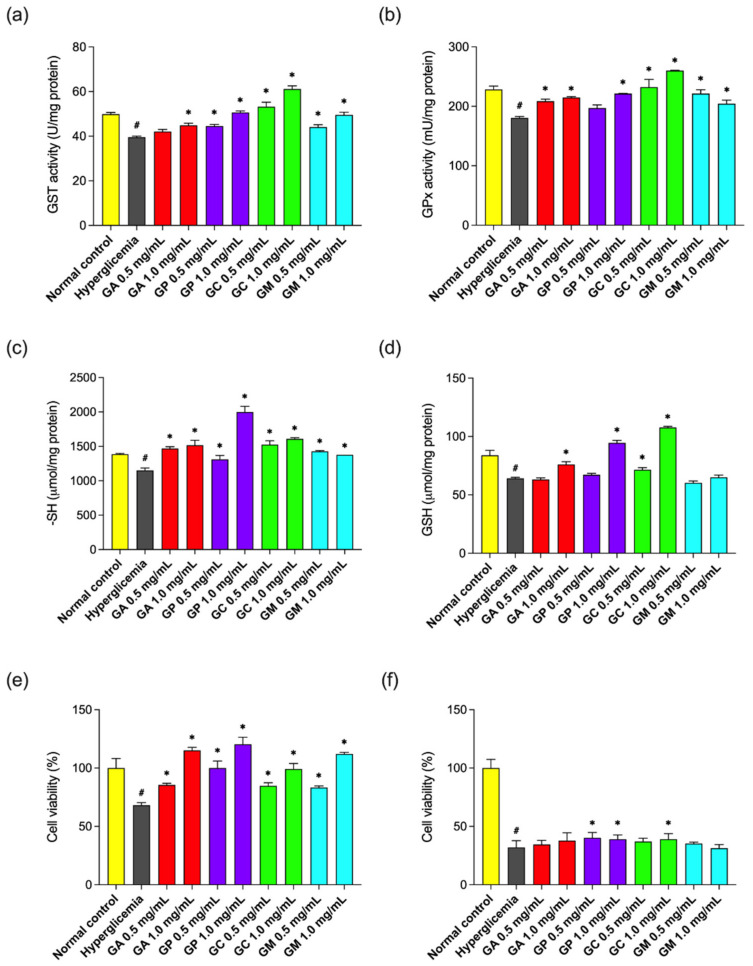
Effect of *G. alypum* (*GA*), *G. punctata* (*GP*), *G. cordifolia* (*GC*) and *G. meridionalis* (*GM*) methanolic leaf extracts on oxidative stress biomarkers in Hep G2 cells cultured under hyperglycemic conditions: (**a**) glutathione S-transferase (GST) activity (U/mg protein); (**b**) glutathione peroxidase (GPx) activity (mU/mg protein); (**c**) free thiol groups (-SH) content (μmol/mg protein); (**d**) reduced glutathione (GSH) content (μmol/mg protein); (**e**) cell viability (%) assessed by the lactate dehydrogenase (LDH) assay; (**f**) cell viability (%) assessed by the 3-(4,5-dimethylthiazol-2-yl)-2,5-diphenyltetrazolium bromide (MTT) assay; Normal control: cells cultured in medium supplemented with 5.56 mM glucose; Hyperglycemia: cells cultured in medium supplemented with 20 mM glucose; # statistically significant difference in comparison to normal control (*p* < 0.05); * statistically significant difference in comparison to cells cultured in hyperglycemic conditions (*p* < 0.05); values are means + SD (*n* = 3; exceptionally, in the MTT assay, *n* = 8).

In the present study, effects of two different concentrations (0.5 and 1.0 mg/mL) of *Globularia* leaf extracts on biomarkers of oxidative stress have been evaluated in Hep G2 cells cultured under hyperglycemic conditions (Figure 5). Treatments with leaf extracts of all tested species significantly increased GST (13.4–54.5%) and GPx (13.1–44.0%) activities at the higher concentration used (*p* < 0.05). Increased GSH content was observed for *G. alypum* (+18.6%), *G. punctata* (+47.4%), and *G. cordifolia* extracts (+68.1%) at 1.0 mg/mL concentration and *G. cordifolia* extract at 0.5 mg/mL (+11.7%) (*p* < 0.05). All tested samples significantly increased free thiol groups content (+14.0–73.7%) and cell viability in the LDH assay (+22.2–76.7%) (*p* < 0.05), while in the MTT assay, a 21.6–25.4% increase was observed only for *G. punctata* (both concentrations) and *G. cordifolia* (c = 1.0 mg/mL) (*p* < 0.05). Overall, *G. cordifolia* reduced the pro-oxidant effects of hyperglycemic conditions observed in Hep G2 cells in all performed assays. Its favorable effect on oxidative status (elevated SOD, CAT and GSH) has already been recorded in human keratinocytes [60].

Extract of *G. cordifolia* was characterized by the highest condensed tannin content (Table 1). Correlation analysis revealed excellent positive correlation between GST activity and condensed tannin content (*r =* 0.94, *p* < 0.001), and very good positive correlation between GPx activity and condensed tannin content (*r =* 0.79, *p* < 0.05) (Table S2). Condensed tannin supplementation of ruminants’ diets was seen to improve their antioxidant status both directly and indirectly [61]. Very good positive correlation was also observed between GST activity and iridoid content (*r =* 0.73, *p* < 0.05) (Table S2). The major common iridoid for *G. cordifolia*, *G. meridionalis* and *G. punctata* (Figure 1, Table 3), which were observed to induce the GST activity more strongly in comparison to *G. alypum* (Figure 5), was asperuloside. This compound was reported for its anti-inflammatory activity by reducing lipopolysaccharide (LPS)- and interferon-γ-(IFN-γ) stimulated nitric oxide (NO) production in RAW264.7 macrophage cells [62]. Increased levels of colon sulfhydryl groups and induced SOD and GPx activities were reported in rats with acetic acid-induced ulcerative colitis [48] as well as with loperamide-induced constipation [63] that were pre-fed with aqueous leaf extract of *G. alypum*, while a protective effect of G*. alypum* methanolic leaf extract via improvement of enzymatic and non-enzymatic antioxidants was observed in rats with deltamethrin-induced nephrotoxicity. The latter extract prepared by maceration was rich in phenylethanoids and flavones [37], many of which were found in *G. alypum* leaf extract obtained by ultrasound-assisted extraction (Table 3).

Very good positive correlation was found between cell viability assessed by the LDH assay and flavonoid content (*r =* 0.88, *p* < 0.01) as well as total phenolic content (*r =* 0.75, *p* < 0.05) (Table S2). According to previous studies, flavonoids and phenylethanoids are the main constituents of the *Globularia* species that possess direct antioxidant activity [29,35,64,65]. The same compounds may be responsible for the direct protection of the cell membrane from oxidative damage, which could have led to the observed improved cell viability, which was subsequently verified by the use of the DPPH assay. Phenylethanoids present in the investigated *Globularia* species, e.g., verbascoside [64,65], rossicaside A, trichosanthoside A, trichosanthoside B [64], leucosceptoside A, and calceolarioside A [65] were recognized as effective scavengers of the DPPH free radical. Some of these compounds were found exclusively either in G*. alypum* or in *G. punctata* (Table 3). The latter species, which showed the greatest protective effect in the LDH assay (Figure 5), contained the most 6-hydroxyluteolin glycosides, which were observed in *G. alypum* as the more potent DPPH radical scavengers (IC_50_ = 6.6–12.2 µM) than its phenylethanoids (IC_50_ =11.8–15.5 µM) and iridoids (IC_50_ = 28.2–76.0 µM), as well as than quercetin (IC_50_ = 7.8 µM) and butylated hydroxytoluene (IC_50_ = 30.0 µM) [35]. Verbascoside was recently reported to protect Smulow–Glickman (S–G) gingival epithelial cells against glucose-induced oxidative stress, which may indicate its potential to improve impaired oral wound healing in diabetic patients [66]. In the same study, it was also observed to enhance mitochondrial function and improve cell survival (50 and 100 µM) in both the LDH assay and the MTT assay.

#### 2.3.3. DPPH Radical Scavenging Activity

Direct antioxidant activity of studied species was confirmed by the DPPH assay, which is the most frequently used assay for evaluation of antioxidant activity of *G. alypum* [11,35,37,47,57,67,68,69] and related species of the genus *Globularia* [29,30], to enable comparison of results with published data. All four species showed good antiradical activity against the DPPH free radical (Table 5). The IC_50_ values ranged from 17.25 μg/mL (*G. alypum*) to 24.19 μg/mL (*G. punctata*). The obtained results are in accordance with our previous study, in which relatively higher antioxidant activities of both *G. alypum* leaf and flower extracts and relatively lower antioxidant activity of *G. punctata* flower extract were observed [23]. Obtained IC_50_ value for *G. alypum* is comparable to previous reports for the diethyl ether fraction of its aerial parts (IC_50_ = 20.54 μg/mL) [47], methanolic extracts of its leaves (IC_50_ = 15.58–27.54 μg/mL [57], IC_50_ = 25.65 μg/mL) and stems (IC_50_ = 22.11 μg/mL) [68], and ethanolic extract of its aerial parts (IC_50_ = 23.50 μg/mL) [69]. Similarly, the IC_50_ value established for *G. meridionalis* is in accordance with that previously reported for methanolic extract of its aerial parts obtained by maceration (IC_50_ = 21.00 μg/mL), from whose methanolic fraction verbascoside (acteoside), isoverbascoside (isoacteoside) and apigenin 7*-O-*glucoside were isolated [29]. Excellent negative correlation was observed between the obtained IC_50_ values for DPPH radical scavenging activity and the estimated total phenolic content of aerial parts extracts (*r =* −0.96, *p* < 0.05) (Table S2), which is in accordance with previous reports [23,68]. Potent anti-DPPH activity comparable to that of ascorbic acid was reported for different phenylethanoids, including calceolarioside A (desrhamnosyl acteoside) (IC_50_ = 22.9 µM) and calceolarioside B (desrhamnosyl isoacteoside) (IC_50_ = 26.2 µM) [70], found only in the phenolic compound-rich extract of *G. alypum*.

To identify the constituents responsible for observed activity, TLC bioautography with DPPH used as spray reagent was performed as in earlier studies of antioxidant activity of different *Globularia* species’ constituents [64,65,67]. Based on our previous findings that indicated possible contribution of flavonoids and other phenolic compounds to the observed antioxidant effects of *Globularia* [23], as well as literature data on antioxidant activity of isolated constituents of *G. alypum* [35,67], a mobile phase suitable for separation of flavonoid glycosides and phenolic acid derivatives was used [71]. Two dominant yellow zones with Rf = 0.29 and Rf = 0.66, showing prominent antiradical activity, were observed in all four species (Figure S1). The latter was identified as the caffeoyl phenylethanoid glycoside verbascoside in comparison to its reference standard. The same compound was recently reported to possess greater DPPH radical scavenging capacity (IC_50_ = 58.1 µM) than ascorbic acid [72]. The former compound was tentatively identified as its glycosylated derivative rossicaside A based on its blue fluorescence at 365 nm observed after NP/PEG treatment and recorded presence in all studied species (Figure S2), as well as earlier reports of its presence in these and related *Globularia* species [22,30,33,37,39,40,64]. Conversely to the previous method in which *G. punctata* showed the lowest radical scavenging activity, the second method revealed at least three zones characteristic for this species showing prominent antiradical activity (Rf = 0.23, Rf = 0.37 and Rf = 0.42). Their Rf values matched those of three orange, fluorescent zones observed at 365 nm after NP/PEG treatment, presumably of flavone origin [71]. Based on comparison to the LC-MS profile of *G. punctata* (Table 4) and literature reports on the flavones isolated from *Globularia* elongata Hegetschw. (syn. *G. punctata*) [42], they were tentatively identified as 6-hydroxyluteolin 7*-O-*sophoroside, 6-hydroxyluteolin 7*-O-*(6′′′*-O-*caffeoyl)-sophoroside, and 6-hydroxyluteolin 7*-O-*(6′′′*-O-*(*p-*coumaroyl))-sophoroside.

### 2.4. Anti-Inflammatory Potential

Cyclooxygenase isoenzymes COX-1 and COX-2 are first in the cascade of enzymes responsible for metabolism of arachidonic acid to prostaglandins (PGE_2_, PGD_2_, PGF_2α_, and PGI_2_) and thromboxane A_2_ [73]. Due to having two functionally coupled active sites, they catalyze conversion of arachidonic acid first to PGG_2_ (cyclooxygenase site), which is afterwards reduced to PGH_2_ (peroxidase site) [74]. Although COX-1 is usually considered the constitutively expressed isoform in most tissues, its increased expression was observed in several human cancers, including ovarian and breast cancer [73]. Anti-inflammatory potential of *G. alypum*, *G. punctata*, *G. cordifolia* and *G. meridionalis* methanolic extract of aerial parts obtained by Soxhlet extraction was evaluated spectrophotometrically using two methods, one based on inhibition of peroxidase activity of COX-1 using *N*,*N*,*N*′,*N*′-tetramethyl-*p*-phenylenediamine dihydrochloride (TMPD assay) and the other based on inhibition of its cyclooxygenase activity assessed by the prostaglandin E_2_ assay (PGE_2_ assay). All tested species inhibited COX-1 activity (Table 6). Extract (c = 50 μg/mL) inhibitory activities ranged from 17.6% (*G. meridionalis*) to 51.3% (*G*. *alypum*) in the TMPD assay (IC_50_ = 2.90 μM for indomethacin), and from 25.7% (*G. meridionalis*) to 40.6% (*G. alypum*) in the PGE_2_ assay (IC_50_ = 1.03 μM for indomethacin). The results obtained for *G. alypum* are comparable to those reported for methanolic leaf (5.33%) and flower extract (61.05%) of the same species tested at 33 μg/mL concentration using the TMPD assay [75].

No correlation between the obtained results and assessed total phenolic or flavonoid content was found (Table S2). Inhibitory activity against COX-1 of verbascoside (IC_50_ > 1 mM), one of the major metabolites found in all investigated species, was lower than that against COX-2 (IC_50_ = 0.69 mM) [76]. Among the isolated compounds of *Anisomeles indica* (L.) Kuntze, of which verbascoside, isoverbascoside, calceolarioside A and apigenin were also recorded in investigated *Globularia* extracts, the latter compound gave the lowest docking score with COX-1 (−6.558) and COX-2 receptors (−8.441) in molecular docking analysis, which indicated its strong binding to the enzymes’ active sites [77]. Based on our previous study [22], apigenin and its glycosides seem to be more common for *G. alypum*, for which the tendency of greater COX-1 inhibition was observed. Anti-inflammatory potential of investigated species could also be connected to their possible diminishing effects on other pro-inflammatory mediators (e.g., NO), and cytokines such as interleukin-1β (IL-1β), interleukin-6 (IL-6) and tumor necrosis factor-α (TNF-α) [78,79,80,81].

Catalpol, the iridoid present together with its many esters and/or derivatives (including globularin) in *G. alypum* and *G. punctata*, was recently reported to protect against glucose-induced podocyte injury by ameliorating apoptosis and inflammation through reduction of TNF-α, IL-6 and IL-1β at 1–10 μM concentrations, which would suggest its preventive potential against diabetic nephropathy [78]. The same compound showed potentially useful effects in other experimental diabetic complications, glucose-lowering effect in experimental T1DM and T2DM possibly due to improved glucose utilization in insulin-sensitive tissues and improved mitochondrial biogenesis/function, as well as neuroprotective and cardioprotective effect, which are all potentially linked to its antioxidant and anti-inflammatory activities [79]. Similarly, verbascoside was reported to reduce the glucose-induced production of inflammatory cytokines, IL-6 and IL-1β in Smulow-Glickman gingival epithelial cells exposed to high glucose concentrations [66], as well as to reduce cellular inflammation and improve mitochondrial activity and survival of pancreatic β-cells under endoplasmic reticulum-stress [82]. Asperuloside and asperulosidic acid, which were observed in *G. punctata*, *G. cordifolia* and *G. meridionalis*, significantly decreased the production of NO, PGE_2_, TNF-α, and IL-6, and inhibited the expression of inducible NO synthase (iNOS), COX-2, TNF-α, and IL-6 mRNA in LPS-induced RAW 264.7 cells [80]. On the other hand, globularifolin (10*-O-*benzoyl-monomelittoside), the major metabolite of *G. cordifolia* and *G. meridionalis*, was reported to significantly suppress expression of nuclear factor-κB (NF-κB) in LPS-stimulated cells at 200 μM concentration [26]. Reduction of IL-6 and TNF-α was also reported for liriodendrin [81], a lignan diglucoside detected in *G. alypum*. The same compound, together with other above-mentioned compounds (e.g., asperuloside, globularin, catalpol, verbascoside), as well as other major compounds identified in this study (e.g., rossicaside A, trichosanthoside A, trichosanthoside B, globuloside A), were also reported for *G. trichosantha* [30,64,83], whose roots/above ground parts (decoction) or leaves (externally) are traditionally used for hemorrhoid treatment in Turkey [84], which may be explained by its inflammation-soothing properties for which these compounds could be accountable.

### 2.5. Antibacterial Potential

*Staphylococcus aureus*, a Gram-positive bacterium, is the main causative pathogen in diabetic foot infections, with methicillin-resistant *S. aureus* (MRSA) being the major multidrug resistant bacterium. Other pathogens may include Gram-positive bacteria, such as *Streptococcus* spp. and *Enterococcus faecalis*, and Gram-negative bacteria, such as *Pseudomonas aeruginosa*, *Escherichia coli*, *Proteus* spp., *Enterobacter* spp., and *Klebsiella* spp. [85,86]. Antibacterial potential of *G. alypum*, *G. punctata*, *G. cordifolia* and *G. meridionalis* was evaluated by testing the effect of methanolic extracts of aerial parts obtained by Soxhlet extraction against four Gram-positive (*S. aureus* ATCC 6538, *E. faecalis* ATCC 29212, *Bacillus cereus* ATCC 11778, *B. subtilis* ATCC 6633) and three Gram-negative bacterial strains (*P. aeruginosa* ATCC 27853, *E. coli* ATCC 10536, *K. pneumoniae* MFBF 10402) using two complementary methods; well diffusion and serial broth microdilution method. In the first method, all four species showed notable inhibitory activity against *S. aureus*, with the greatest zone of inhibition recorded for *G. alypum* (25.0 mm), as well as low inhibitory activity against *B. cereus* (Table 7).

Furthermore, moderate to good inhibition against *E. faecalis* and *E. coli* was observed for *G. punctata*, while *G. alypum* and *G. cordifolia* showed low inhibitory activities against *B. subtilis*. Finally, low inhibition against *P. aeruginosa* was also observed for *G. alypum*. Antibacterial effects of *G. alypum* methanolic extracts against *S. aureus* [68,87,88,89], *P. aeruginosa* [88,89], *B. cereus* [88], and *B. subtilis* [68] have already been reported. As in our study, the species demonstrated the strongest antibacterial effect against *S. aureus* [87,88,89,90].

The results of the serial broth microdilution method followed by sub-cultivation on agar plates mainly coincided with those of the first method (Table 8). Assessed MIC values against *S. aureus* ATCC 6538 ranged between 1.42 mg/mL (*G. alypum*, *G. punctata*) and 2.84 mg/mL (*G. cordifolia*, *G. meridionalis*). Low inhibitory activity of *G. alypum* against *P. aeruginosa* (MIC = 22.73 mg/mL) and moderate to good bactericidal/inhibitory activity of *G. punctata* against *E. faecalis* (MBC = 5.68 mg/mL) and *E. coli* (MIC = 1.42 mg/mL) were also confirmed. *C*omparable antibacterial activity *of G. alypum* methanolic extract against *S. aureus* (MIC = 2–4 mg/mL) was reported by previous studies [68,91], while greater inhibitory activity was recorded against *P. aeruginosa* (MIC = 8 mg/mL) [91].

Opposite to the results of the diffusion method, in the second method, *G. punctata* showed good antibacterial activity against *B. cereus* (MIC = 2.84 mg/mL) and *B. subtilis* (MIC = 1.89 mg/mL), while much lower inhibition against *B. cereus* was observed for related species (MIC = 11.36 mg/mL). The latter method possibly accounted for the antibacterial activity of less hydrophilic compounds of *G. punctata*, which might not have been observed earlier due to their lower solubility and consequential lower diffusion into the hydrophilic (agar) medium [92]. Excellent negative correlation (*r* = −0.998, *p* < 0.01) was found between MIC values against *B. cereus* and flavonoid content (Table S2), indicating that the higher anti-*Bacillus* activity observed for *G. punctata* could be connected to its characteristic flavonoid compounds, such as the detected acylated derivatives of 6-hydroxyluteolin 7-*O*-sophoroside. Inhibitory activity against Gram-positive bacteria *B. subtilis*, *B. cereus* and *S. aureus* was reported for flavonoids observed in all four *Globularia* species, luteolin 7-*O*-glucoside [22] (MIC = 50–90 μg/mL) and apigenin (Table 4) (MIC = 30–80 μg/mL), with the latter also showing inhibitory activity against *E. coli* and *P. aeruginosa* [93]. The reported MIC values for apigenin against *B. subtilis*, *E. faecalis* and *P*. *aeruginosa* (the same ATCC strains as used in the present study), were equal to 8, 8 and 64–400 μg/mL, respectively, while against *S. aureus* no inhibitory activity was observed [94].

Nine additional *S. aureus* strains, including three clinical isolates of methicillin-susceptible *S. aureus* (MSSA) and five clinical isolates of MRSA, were also tested (Table 8). Observed MIC values ranged between 1.42 and 11.36 mg/mL, with no significant differences established between MSSA and MRSA strains (*p* > 0.05). Keeping in mind that these are the most common causative pathogens found in diabetic foot infections [7], the observed inhibitory activities can partially explain the reported traditional use of *G. alypum* in the treatment of diabetes-associated foot ulcers. The anti-staphylococcal activity could partly be attributed to verbascoside, for which the assessed inhibitory activity (MIC = 227.2 μg/mL) corresponded to values previously reported against five MRSA strains (MIC = 64–256 μg/mL) [95]. Comparable inhibitory activity against *S. aureus* of all investigated species may also indicate contribution of other common constituents, such as isoverbascoside, rossicaside A, forsythoside A and 6-hydroxyluteolin 7-*O*-glucoside. Verbascoside (MIC = 60 μg/mL) was observed to have greater anti-staphylococcal activity than isoverbascoside (MIC = 130 μg/mL), while both compounds possessed lower inhibitory activities against *E. faecalis* (MIC = 100–150 μg/mL), *E. coli* (MIC = 100–250 μg/mL) and *P. aeruginosa* (MIC = 250 μg/mL) [96]. Other phenylethanoids, such as calceolarioside A and calceolarioside B, characteristic for *G. alypum*, also possess antibacterial potential against Gram-positive and Gram-negative bacteria [97], which might explain the observed inhibitory effect of *G. alypum* against *P. aeruginosa*. The antibacterial potential of *G. punctata* against *E. coli* and *E. faecalis*, as well as *S. aureus*, may be connected to its characteristic phenylethanoids (trichosanthoside A, trichosanthoside B, and arenarioside) and/or flavonoids. Excellent negative correlations were found between flavonoid content and MICs against MSSA MFBF 505 (*r* = −0.960, *p* < 0.05) and MRSA MFBF 154 (*r* = −0.998, *p* < 0.01).

### 2.6. Anticancer Potential

**Figure 6:**
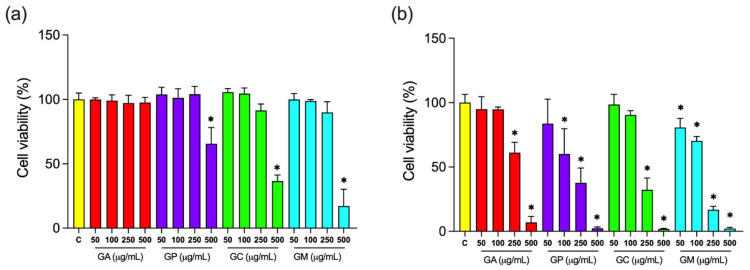
Effect of *G. alypum* (*GA*), *G. punctata* (*GP*), *G. cordifolia* (*GC*) and *G. meridionalis* (*GM*) methanolic leaf extracts on cell viability of: (**a**) MDA-MB-231 breast cancer cell line; (**b**) A1235 glioblastoma cell line; * statistically significant difference in comparison to control (*p* < 0.05); values are means + SD (*n* = 4).

The most common cancer type in the female population both in Europe and in the US, which, depending on the age group and/or country of origin, accounts for the greatest or second greatest number of cancer deaths in women, is breast cancer [98,99]. MDA-MB-231 cells represent the claudin-low, triple-negative subtype of breast cancer that shows intermediate response to chemotherapy and is often associated with poor prognosis [100]. Glioblastoma is another highly aggressive and invasive tumor, classified as the WHO grade IV astrocytoma, which represents 15–20% of all primary intracranial neoplasms in adults. Deaths from glioblastoma usually occur within the first 15–16 months after diagnosis, while the 5-year survival rate is only 5% [101]. Anticancer potential of methanolic leaf extracts of investigated *Globularia* species was evaluated using the MTT assay based on their effects against MDA-MB-231 breast cancer cell line and A1235 glioblastoma cell line. Cytotoxic effect against MDA-MB-231 cell line was observed for extracts of *G. punctata* (34.48%), *G. cordifolia* (63.41%) and *G. meridionalis* (82.96%) (at the highest concentration used), while cytotoxic effect against A1235 cell line was observed for all investigated species (*p* < 0.05) (Figure 6). Greatest cytotoxic effect against A1235 cells was observed for *G. meridionalis* (IC_50_ = 129.40 μg/mL) and the lowest for *G. alypum* (IC_50_ = 231.43 μg/mL) (Table 9).

The observed cytotoxic effects against MDA-MB-231 cells that were shown by *G. meridionalis*, *G. cordifolia* and *G. punctata* may be connected to asperuloside and its derivatives (Table 3) [102,103,104]. Ethanolic extract of *Oldenlandia diffusa* (Willd.) Roxb. that contained asperuloside and deacetylasperulosidic acid also reduced MDA-MB-231 and MDA-MB-453 cell viability and suppressed their colony formation capacities [102]. Cytotoxic effect against YMB-1 breast cancer cells (IC_50_ = 0.7 μg/mL), HL60 cells (IC_50_ = 11.0 μg/mL) and KB cells (IC_50_ = 104.2 μg/mL) was recorded for asperuloside [103], while asperulosidic acid reduced the viability of HT-29 human colon adenocarcinoma cells (IC_50_ = 6.1 μg/mL) [104]. Globularifolin, whose cytotoxic activity against CAMA-1 breast cancer cells (IC_50_ = 10 μM) was reported [28], could be responsible for the more pronounced inhibition of MDA-MB-231 cell viability recorded for *G. cordifolia* and *G. meridionalis*. Very good negative correlations were established between observed cell viability and concentrations of flavonoids (*r* = −0.72, *p* < 0.01), iridoids (*r* = −0.75, *p* < 0.001), and total phenolics (*r* = −0.81, *p* < 0.001), while excellent correlation was found between MDA-MB-231 cell viability and concentration of condensed tannins (*r* = −0.90, *p* < 0.001) (Table S2). Cytotoxic effect of condensed tannins was reported against MCF-7 and Hs 578T breast cancer cells, Caco-2 colon carcinoma cells, and DU 145 prostate cancer cells [105]. Cytotoxic effect against MDA-MB-231 (IC_50_ = 0.159–0.258 μM) [106] and a range of other cancer cell lines, including U87 glioblastoma cell line, was reported for verbascoside [107], the major phenylethanoid of investigated species.

Moreover, investigation of cytotoxic activity of *Callicarpa nudiflora* Hook. and Arn. against HeLa cells, A549 lung adenocarcinoma cells and MCF-7 cells resulted in isolation of flavonoids, phenylethanoids and iridoids, which included those found in investigated species, 6-hydroxyluteolin 7-*O*-glucoside, verbascoside and catalpol [108]. Weak cytotoxic effect against HeLa cells (IC_50_ = 1530 μg/mL) was also reported for *G. alypum* [109].

Excellent negative correlations were established between A1235 cell viability and concentrations of total phenolics (*r* = −0.95, *p* < 0.001), flavonoids (*r* = −0.90, *p* < 0.001) and condensed tannins (*r* = −0.87, *p* < 0.001), while very good negative correlation was observed between A1235 cell viability and iridoid concentrations (*r* = −0.78, *p* < 0.001) (Table S2). Common phenolic compounds included verbascoside, isoverbascoside, rossicaside A, forsythoside A, leucosceptoside A, globusintenoside isomer, and 6-hydroxyluteolin 7-*O*-glucoside, while in *G. punctata*, *G. cordifolia* and *G. meridionalis* benzoyl-rossicaside A isomer was also found. Investigations conducted on different cancer cell lines indicated that rhamnose may be important for cytotoxic activity of phenylethanoids [110,111]. This could explain the observed relatively lower cytotoxic effect of *G. alypum*, whose leaf extract contained a relatively great share of phenylethanoids that do not contain rhamnose in their structure, such as calceolarioside A, calceolarioside B, 6′-*O*-feruloyl-1′-*O*-hydroxytyrosyl glucoside, desrhamnosyl 6′-*O*-caffeoylverbascoside, and desrhamnosyl galypumoside B. On the other hand, the observed cytotoxic effect of *G. cordifolia* and *G. meridionalis* extracts against A1235 cells may again be partially attributed to globularifolin, their common iridoid, which was reported to exert anticancer effects against U87 cells [27].

## 3. Materials and Methods

### 3.1. Chemicals and Reagents

Adrenalin bitartrate and indomethacin were purchased from Acros Organics, Geel, Belgium; glacial acetic acid from Alkaloid, Skopje, North Macedonia; asperuloside and catalpol from Carl Roth, Karlsruhe, Germany; hydrochloric acid from Carlo Erba, Emmendingen, Germany; arachidonic acid from Cayman Chemical Company, Ann Arbor, MI, USA; (+)-catehin, 2,2-diphenyl-1-picrylhydrazyl (DPPH), diphenylboric acid-β-ethylamino ester (natural products reagent, NP) and LC-MS grade formic acid from Fluka, Buchs, Schwitzerland; fetal bovine serum (FBS), minimum essential medium (MEM), penicillin-streptomycin, and trypan blue from Gibco, Gaithersburg, MD, USA; absolute ethanol from Gram-Mol, Zagreb, Croatia; verbascoside from HWI Analytik, Rülzheim, Germany; ethyl acetate, Folin-Ciocalteu’s reagent, D-(+)-glucose, polyethylene glycol 4000 (PEG 4000), potassium chloride, potassium phosphate monobasic, sodium carbonate decahydrate, sodium hydroxide, sodium phosphate dibasic heptahydrate, sulfuric acid and Tween from Kemika, Zagreb, Croatia; gentamicin sulfate and norfloxacin from Krka, Novo mesto, Slovenia; chloroform, copper(II) sulfate pentahydrate, gallic acid, LC-MS grade acetonitrile, methanol, Müller–Hinton agar, Müller–Hinton broth, sodium chloride and Tryptic soy agar (TSA) from Merck, Darmstadt, Germany; Tris-HCl buffer from Santa Cruz Biotechnology, Dallas, TX, USA; formic acid from Scharlau, Scharlab, Barcelona, Spain; acarbose, aluminum chloride hexahydrate, aucubin, 1-chloro-2,4-dinitrobenzene (CDNB), cyclooxygenase-1 (COX-1) from sheep, dimethyl sulfoxide (DMSO), 3-(4,5-dimethylthiazol-2-yl)-2,5-diphenyltetrazolium bromide (MTT), disodium ethylenediamine-tetraacetic acid (Na_2_EDTA), 5,5′-dithiobis(2-nitrobenzoic acid) (DTNB, Ellman’s reagent), Dulbecco’s modified Eagle’s medium (DMEM), Type I α-glucosidase (isolated from *Saccharomyces cerevisiae*), glutathione (GSH), hematin, 4-(2-hydroxyethyl)piperazine-1-ethanesulfonic acid (HEPES), *p-*nitrophenyl-α-d-glucopyranoside (PNG), quercetin, *N,N,N′,N′*-tetramethyl-*p-*phenylenediamine dihydrochloride (TMPD), trichloroacetic acid, 2,3,5-triphenyltetrazolium chloride (TTC), trypsin-EDTA and vanillin from Sigma-Aldrich, St. Louis, MO, USA; and sodium diethyldithiocarbamate trihydrate (DDC) from VWR International, Radnor, PA, USA.

### 3.2. Plant Material

Aerial parts of investigated *Globularia* species were collected during their flowering stage from wild populations in four sampling locations: Konavle cliffs (*G. alypum*, March 2013), Grobnik field (*G. punctata*, May 2012 and 2013, *G. meridionalis*, May 2013), Baške Oštarije, Velebit (*G. cordifolia* and *G. meridionalis*, June 2012) and Alan, Velebit (*G. cordifolia*, May 2013). Plant material was identified by Prof. Kroata Hazler Pilepić. Voucher specimens are deposited in the Herbarium of the Department of Pharmaceutical Botany of the Faculty of Pharmacy and Biochemistry, University of Zagreb. Collection data are provided in Table S3. The plant material was air-dried at room temperature.

### 3.3. Sample Preparation

Ultrasound-assisted extraction of powdered leaves (0.5–1.25 g) was performed using methanol (1:10) for 2 × 30 min. Obtained liquid extracts were filtered and evaporated to dryness using a rotavapor at 50 °C. Soxhlet extraction of powdered aerial parts (145–280 g) was performed using methanol (1:5) for 8 h. Obtained extracts were evaporated to dryness using a rotavapor at 30 °C, re-suspended in 50% (*v/v*) methanol and extracted three times with chloroform. After chloroform removal, the remaining solvents were again evaporated at 30 °C and freeze-dried. All samples were stored at −20 °C until further use.

### 3.4. Phytochemical Content

Phytochemical content was evaluated spectrophotometrically as described previously [23]; total phenolic content using the Folin–Ciocalteu assay, flavonoid content using the aluminum chloride assay, iridoid content using the Trim–Hill assay, and condensed tannin content using the vanillin assay. Results are expressed as gallic acid equivalents (GAE)/g dry extract (DE), quercetin equivalents (QE)/g DE, aucubin equivalents (AE)/g DE, and catechin equivalents (CE)/g DE, respectively.

### 3.5. HPLC-PDA-ESI-MS^n^ Analysis

High-performance liquid chromatography-photodiode array detection-electrospray ionization-tandem mass spectrometry, HPLC-PDA-ESI-MS^n^, using the LTQ XL linear ion trap mass spectrometer coupled to the Dionex UltiMate 3000 liquid chromatograph and photodiode array detector (Thermo Fisher Scientific, Waltham, MA, USA), was used for the analysis of methanolic leaf and aerial parts extracts composition. Zorbax SB-C_18_ column (i.d. 150 mm × 2.1 mm, 3.5 μm) (Agilent Technologies, Santa Clara, CA, USA) was used as a stationary phase. Separation of constituents and data collection were carried out under the previously described conditions [22]. UV/Vis spectral data were collected within the range 190–600 nm and mass spectral data within the range *m/z* 50–2000. Electrospray ionization (ESI) was performed in negative ionization mode. MS^n^ spectra (MS^2^–MS^4^) were obtained by collision induced dissociation (CID) of the ion of the greatest intensity in the mass spectrum of lower order with helium as the collision gas and normalized collision energy set at 35%. Data acquisition and processing were performed using Thermo Xcalibur 2.2 (Thermo Fisher Scientific, Waltham, MA, USA). Compound identification was based on the comparison of chromatographic and spectral data to those of previously identified constituents found in methanolic extracts of aerial parts of the same *Globularia* species obtained by heating under reflux conditions [22].

### 3.6. TLC Analysis

Constituents of methanolic aerial parts extracts obtained by Soxhlet extraction (c = 50 mg/mL) were separated on thin-layer chromatography (TLC) silica gel 60 F_254_ aluminum plates (Merck, Darmstadt, Germany), 10 × 20 cm, thickness 0.20 mm, using chloroform-methanol-wate*r =* 61:32:7 (*v/v/v*), or ethyl acetate-methanol-wate*r =* 20:2:1 (*v/v/v*) as the mobile phase [36]. Sample and standard solutions (10 μL) were applied 1.5 cm from the lower edge of the plate. The distance travelled by the mobile phase was 7 cm. Compound identification was based on the comparison of zone color and position observed under white light, UV 365 nm and UV 254 nm (Camag Reprostar 3, Muttenz, Switzerland) after treatment with 1% (*w/v*) ethanolic vanillin-5% (*v/v*) ethanolic sulfuric acid reagent (5–10 min at 100–105 °C), to those of asperuloside, aucubin and catalpol reference standards (c = 0.5 mg/mL).

### 3.7. Assessment of Antidiabetic and Antioxidant Potential

#### 3.7.1. α-Glucosidase Activity

Inhibition of α-glucosidase activity was assessed using PNG as previously described [112]. Methanolic leaf extract solutions (100 µL) in 2% (*v/v*) DMSO were mixed with 50 µL Type I α-glucosidase from *Saccharomyces cerevisiae* (1.0 U/mL in 0.1 M phosphate buffer, pH 6.8) and preincubated at 37 °C. After 10 min, 50 µL PNG (5 mM in 0.1 M phosphate buffer, pH 6.8) was added. Absorbance was measured for 5 min at 405 nm against a blank in which PNG was replaced with phosphate buffer. Results are expressed as enzyme activity % in comparison to control (2% (*v/v*) DMSO), which was considered to give 100% enzyme activity. Acarbose was used as positive control.

#### 3.7.2. Cell Culture and Treatment

Hep G2 cells obtained from European Collection of Authenticated Cell Cultures (ECACC, Salisbury, UK) were cultured in MEM supplemented with 10% (*v/v*) FBS, 20 IU/mL penicillin and 20 µg/mL streptomycin at 37 °C and 5% CO_2_. After removal of culture medium, cells were washed with phosphate buffered saline (PBS), trypsinized using 0.25% trypsin-EDTA solution for 5 min at 37 °C, counted under a light microscope (Leitz Diavert) after 0.04% trypan blue staining using the Bürker–Türk counting chamber and seeded into six-well plates (1.67 × 10^6^) with FBS-free medium or 96-well plates (2 × 10^5^) (MTT assay). After 24 h incubation at 37 °C, cells were washed with PBS and incubated with methanolic leaf extract solutions (c = 0.5 and 1.0 mg/mL) in MEM with 20 mM glucose (hyperglycemic conditions) for 24 h at 37 °C. Normal control cells were kept in MEM with 5.56 mM glucose. Prior to cell treatment, samples were filtered using sterile Nalgene filter units of pore size 0.2 μm (Sigma-Aldrich, St. Louis, MO, USA). After incubation and washing with PBS, the cells were lysed with 1% (*v/v*) Tween in PBS with the help of ultrasound (4 W) (Cole-Parmer 4710) for 15 s and centrifuged for 20 min at 14,000 rpm at 4 °C. The supernatant was stored at −80 °C until further use.

#### 3.7.3. Oxidative Stress Biomarkers and Cell Viability Assessment

Glutathione peroxidase (GPx) activity was measured spectrophotometrically using the Glutathione Peroxidase Activity Colorimetric Assay Kit (Biovision, Milpitas, CA, USA). Glutathione S-transferase (GST) activity was evaluated spectrophotometrically using CDNB as the substrate [113]. The cell lysate supernatant was mixed with 1 mM GSH and 1 mM CDNB in 50 mM HEPES, pH 7.4. Absorbance was measured for 75 s at 340 nm and 37 °C against reagent blank on the semi-automatic analyzer (Trace 30). GST activity was calculated using the GS-DNB molar extinction coefficient at 340 nm (9600 cm^−1^M^−1^). Content of free thiol groups (-SH) was evaluated spectrophotometrically using Ellman’s reagent [114]. Reaction mixture (1000 μL) consisted of cell lysate supernatant (100 μL), 0.25 M Tris-HCl buffer, pH 8.2, with 20 mM EDTA (150 μL), and 10 mM Ellman’s reagent (10 μL) in absolute methanol. After incubation (20 min) and centrifugation (3000 rpm, 10 min), absorbance was measured at 412 nm against distilled water blank (Cecil Aquarius CE 7200). Results were calculated using the molar extinction coefficient of the product (14,150 cm^−1^M^−1^). For evaluation of GSH content, as previously described [112], to deproteinize the cell lysate supernatant (300 µL), 5% (*v/v*) trichloroacetic acid was added (100 µL), and the mixture was centrifuged (3000 rpm, 10 min). To obtained supernatant (100 µL), PBS (550 µL, 0.3 M, pH 7.4) and DTNB (50 µL) dissolved in the same buffer were added. The production of yellow colored 5-thio-2-nitrobenzoic acid (TNB) was measured at 405 nm against reagent blank. Results were calculated using the molar extinction coefficient of the product (14,150 cm^−1^M^−1^). All results are expressed per mg protein. Protein concentrations were established fluorometrically using the Qubit Protein Assay Kit (Invitrogen).

Viability of Hep G2 cells was evaluated spectrophotometrically using the cell lysate supernatant by measuring lactate dehydrogenase (LDH) activity as an indicator of cell membrane damage [115] with commercial reagent. In 15 μL supernatant, 900 μL reagent (100 mM Tris, 7 mM nicotinamide adenine dinucleotide (NAD+), 50 mM lithium lactate and 120 mM KCl, pH 9.0) was added. Absorbance was read after 30 and 60 s at 340 nm and 37 °C (Trace 30) against distilled water blank. Results were calculated using the molar extinction coefficient of NADH at 340 nm (6300 cm^−1^M^−1^). Cell viability was also evaluated based on the assessment of their metabolic/mitochondrial function using the MTT assay as previously described [116]. After 24 h treatment, culture medium was removed and cells were washed two times with PBS and afterwards incubated in MEM (1 mL) with 5 mg/mL MTT solution in PBS (50 μL). After incubation for 4 h at 37 °C, MTT solution was removed and the cells were washed two times with PBS, and the obtained formazan crystals were dissolved in DMSO. Absorbance was measured at 595 nm (Victor3 1420 Multilabel Counter, Perkin Elmer, Waltham, MA, USA). Results are expressed as cell viability %, in comparison to control, which was considered to give 100% cell viability.

#### 3.7.4. DPPH Radical Scavenging Activity Assay

Antiradical activity was assessed using the free radical 2,2-diphenyl-1-picrylhydrazyl (DPPH) according to the previously described procedure [23]. Methanolic stock solution of DPPH (0.1 mM) was dissolved to obtain absorbance of 0.70 ± 0.02. In 2 mL of prepared solution, 10 µL sample was added. After 30 min incubation, absorbance was measured at 517 nm with methanol used as blank. Results are expressed as concentrations observed to reduce the DPPH radical absorbance by 50% (IC_50_).

#### 3.7.5. TLC Bioautography Assay

Constituents of methanolic aerial parts extracts obtained by Soxhlet extraction (c = 20 mg/mL) were separated on TLC silica gel 60 F_254_ glass plates (Merck), 20 × 20 cm, thickness 0.25 mm, using ethyl acetate-formic acid-glacial acetic acid-water (100:11:11:26, *v/v/v/v*) as the mobile phase [71]. Sample and standard solutions (20 μL) were applied 2.5 cm from the lower edge of the plate. The distance travelled by the mobile phase was 15 cm. Plates were sprayed with 0.05% (*w/v*) methanolic solution of DPPH [117]. Constituents possessing antioxidant activity were detected as white to yellow zones against a light purple background. Compound identification was based on the comparison of zone color and position observed under UV 365 nm after treatment with 1% (*w/v*) methanolic diphenylboric acid-β-ethylamino ester, and 5% (*w/v*) ethanolic polyethylene glycol 4000 (NP/PEG reagent) [71], to that of verbascoside reference standard (c = 0.5 mg/mL).

### 3.8. Assessment of Anti-Inflammatory Potential

#### 3.8.1. PGE_2_ Assay

Cyclooxygenase activity of COX-1 was evaluated spectrophotometrically according to previously described protocols [118,119]. COX-1 solution was first diluted in 80 mM Tris-HCl buffer, pH 8.0, with 0.1% (*v/v*) Tween and 300 μM DDC (c = 200 U/mL) and then in 0.1 M Tris-HCl buffer, pH 8.0 (1:100) just before the performed assay, while hematin (c = 1 mM) was dissolved in 0.01 M NaOH and afterwards diluted in 0.1 M Tris-HCl buffer, pH 8.0 (1:10). Sample (methanolic aerial parts extract solution, 10 μL) was preincubated with 0.1 M Tris-HCl buffer, pH 8.0 (20 μL), 72 mM adrenalin bitartrate (50 μL), 2 U/mL COX-1 (100 μL) and 100 μM hematin (10 μL) for 5 min at room temperature. The reaction was started by the addition of 100 μM arachidonic acid (10 μL). After 20 min incubation at 37 °C, the reaction was terminated by the addition of 10% (*v/v*) formic acid (10 μL). Concentration of produced PGE_2_ was evaluated using the Prostaglandin E2 EIA Kit-Monoclonal (Cayman Chemical Company, Ann Arbor, MI, USA). After 18 h sample incubation with PGE_2_ tracer (PGE_2_-acethylcholinesterase conjugate) and PGE_2_ monoclonal antibody at 4 °C, wells were emptied and rinsed five times with wash buffer. After 90 min incubation with Ellman’s reagent, absorbance was read at 405 nm using the iEMS Reader MF type 1401 (Labsystems, Vantaa, Finland) and corrected for the absorbance at 620 nm. Blank absorbance (Ellman’s reagent) and non-specific binding absorbance (absence of antibody) were subtracted from the readings. From absorbances obtained for samples and control (ethanol), PGE_2_ tracer binding % were calculated, which were inversely proportional to the PGE_2_ concentrations in the wells. Results are expressed as COX-1 inhibition %, calculated from established PGE_2_ concentrations. Indomethacin was used as positive control.

#### 3.8.2. TMPD Assay

Peroxidase activity of COX-1 was evaluated spectrophotometrically using the TMPD assay [120], with some modifications. Sample (methanolic aerial parts extract solution, 10 μL) was pre-incubated for 10 min at room temperature with 0.1 M Tris-HCl buffer, pH 8.0 (60 μL), 200 U/mL COX-1 enzyme in 0.1 M Tris-HCl buffer, pH 8.0 (100 μL), 100 μM hematin (10 μL) and 2 mM TMPD in DMSO (10 μL). The reaction was started by the addition of 2 mM arachidonic acid (10 μL). After 20 s, absorbance was measured at 620 nm using the iEMS Reader MF type 1401 (Labsystems, Vantaa, Finland). Blank absorbance (without arachidonic acid) and non-specific absorbance (without COX-1) were subtracted from absorbance readings. Results are expressed as COX-1 inhibition %, obtained from the difference between control (ethanol) and sample absorbance, divided by control absorbance. Indomethacin was used as positive control.

### 3.9. Assessment of Antibacterial Potential

#### 3.9.1. Bacterial Strains and Inoculum and Sample Preparation

Antibacterial activity was evaluated using the following strains: *Bacillus cereus* American Type Culture Collection (ATCC, Manassas, VA, USA) 11778, *B. subtilis* ATCC 6633, *Enterococcus faecalis* ATCC 29212, *Staphylococcus aureus* ATCC 6538 and *S. aureus* ATCC 29213 among Gram-positive and *Escherichia coli* ATCC 10536, *Klebsiella pneumoniae* MFBF 10402 and *Pseudomonas aeruginosa* ATCC 27853 among Gram-negative bacterial species. Additionally, eight clinical isolates of *S. aureus*, including methicillin-resistant *S. aureus* (MRSA MFB F101, MRSA MFBF 124, MRSA MFBF 154, MRSA MFBF 164, and MRSA MFBF 177) and methicillin susceptible *S. aureus* (*S. aureus* MFBF 505, *S. aureus* MFBF 10661 and *S. aureus* MFBF 10666) were used. Bacterial strains were sourced from the Collection of Microorganisms, Department of Microbiology, Faculty of Pharmacy and Biochemistry, University of Zagreb. Inoculums were prepared from overnight cultures that were cultured on tryptic soy agar (TSA) at 37 °C, suspended in sterile physiological saline, with the optic density adjusted to 0.5 McFarland (1.5 × 10^8^ colony-forming units (CFUs)/mL) using a densitometer (Densimat, BioMérieux, Marcy-l’Étoile, France). Right before cultivation in nutrient medium, 1 mL of each prepared suspension was dissolved with 9 mL physiological saline. Prior to cell treatment, sample stock solutions in distilled water (c = 50 mg/mL) were filtered through Chromafil cellulose acetate filters of pore size 0.22 μm (Macherey-Nagel, Düren, Germany).

#### 3.9.2. Well Diffusion Method

Well diffusion method was carried out according to the European Pharmacopoeia [121]. Inoculums (100 μL) were transferred to individual petri dishes with 4 mm thick Müller–Hinton agar and equally distributed across agar surface with a glass L-stick. Wells were prepared using sterile stainless-steel cylinders (*d* = 6 mm) and filled with 50 μL of methanolic aerial parts extract solution (c = 50 mg/mL) in distilled water. Plates were incubated at 37 °C for 18 h in the dark. Diameters of the zones of inhibition of bacterial growth (mm) were read from the diameters of transparent zones around the wells. Gentamicin and norfloxacin (c = 0.2 mg/mL) were used as positive controls.

#### 3.9.3. Serial Broth Microdilution Method with Agar Sub-Cultivation

Broth microdilution method was conducted in sterile plastic microtiter plates with 96 wells (Nuova Aptaca, Canelli, Italy), in accordance with the Clinical and Laboratory Standards Institute (CLSI) recommendations [122]. Two-fold serial dilutions of sample (methanolic aerial parts extract solution) or verbascoside reference standard were prepared in Müller–Hinton broth (100 μL), to which 10 μL diluted inoculum (1:10) was added. Growth control well consisted of 100 μL Müller–Hinton broth and 10 μL diluted inoculum. Minimum inhibitory concentrations (MICs), defined as the lowest concentrations of samples that inhibit the visible growth of microorganisms, were read after 18 h incubation at 37 °C. Due to extract turbidity, to avoid subjectivity of visual turbidity readings, indicative of bacterial growth, MICs were assessed after 3 h incubation at 37 °C with 1% (*w/v*) TTC (20 μL), which in the presence of metabolically active bacteria (except *E. faecalis*) gave red coloration/precipitate [123]. This was performed after agar sub-cultivation, in which content from each well (10 μL) was transferred to the surface of Müller–Hinton agar, divided into eight sectors, with a calibrated inoculation loop, and incubated for 18 h at 37 °C. Minimum bactericidal concentrations (MBCs) were determined as the lowest concentrations of samples that lead to absence of bacterial growth on agar plates. Gentamicin was used as positive control against ATCC bacterial strains.

### 3.10. Assessment of Anticancer Potential

#### 3.10.1. Cell Culture and Treatment

Human MDA MB-231 breast cancer cells obtained from Dr. Sonja Levanat and human A1235 glioblastoma cells obtained from S.A. Aaronson [124] were cultured in DMEM, supplemented with 10% fetal bovine serum, in a humidified atmosphere at 37 °C and 5% CO_2_, as previously described [125]. After removal of culture medium, cells were trypsinized using 0.25% trypsin-EDTA solution for 5 min at 37 °C, counted on Z2 Coulter Counter (Beckman Coulter, Brea, CA, USA) and seeded in 96-well culture plates (5 × 10^3^). Cells were incubated with methanolic leaf extract solutions in DMEM (c = 50, 100, 250 and 500 μg/mL) for 24 h at 37 °C. Prior to cell treatment, samples were filtered through sterile filters of pore size 0.22 μm.

#### 3.10.2. Cell Viability Assay

Cell viability was assessed using the MTT assay as previously described [125]. Prior to addition of MTT dye (0.5 mg/mL) to each well, the medium was removed, and the cells were washed with PBS. After 4 h of incubation at 37 °C, obtained formazan crystals were dissolved in DMSO (170 μL) and plates were agitated for 10 min at 600 rpm. Absorbances were measured at 570 nm using a microplate reader (Victor3 1420 Multilabel Counter, Perkin Elmer, Waltham, MA, USA). Results are presented as cell viability % in comparison to control, which was considered to give 100% cell viability.

### 3.11. Statistical Analysis

Phytochemical content, assessment of oxidative stress biomarkers and viability of Hep G2 cells in hyperglycemic conditions (LDH assay), antibacterial activity and COX-1 inhibitory activity (TMPD assay) were evaluated in triplicate and the results are expressed as mean values and standard deviations or, exceptionally, mean values (serial broth microdilution method, agar sub-cultivation). Assessment of *α*-glucosidase activity, COX-1 inhibitory activity (PGE_2_ assay) and MDA-MB-231 and A1235 cell viability were performed in quadruplicate, and viability of Hep G2 cells in hyperglycemic conditions (MTT assay) in octuplicate, and the results are expressed as mean values and standard deviations. Concentrations that were observed to reduce the DPPH radical absorbance by 50% (IC_50_) were estimated using linear regression and those that reduced COX-1 activity and cell viability by 50% (IC_50_) using logarithmic regression.

Statistically significant differences were evaluated by using one-way analysis of variance (ANOVA), followed by Tukey’s post hoc test (comparison between different species) or Dunnett’s post hoc test (comparison between samples and control/hyperglycemia). Pearson’s correlation coefficient (*r*) was used to establish the relationship between observed biological activities and phytochemical content. In all tests, significance level α was set at 0.05. Analyses were performed using GraphPad Prism 6.01 (GraphPad Software, San Diego, CA, USA).

## 4. Conclusions

The present study provides a greater insight into the phytochemical composition of *G. alypum* and three related species, *G. punctata*, *G. cordifolia*, and *G. meridionalis*, considering different methods of extract preparation and different plant parts used. The bioactive compounds contained in investigated extracts of *G. alypum* and their observed biological activities are in accordance with the results of previous biological activity studies, as well as the reported traditional uses of this well-investigated medicinal plant, including its antidiabetic use, while those of related species suggest they too may have therapeutic potential. Observed antioxidant, anti-inflammatory, and antimicrobial activities of aerial parts extracts of investigated *Globularia* species support their use in cosmetics, while cytotoxicity assay results indicate further studies should preferably be carried out on *G. cordifolia*, *G. meridionalis* and *G. punctata*. The latter species also showed greater antimicrobial potential in comparison to *G. alypum*, which may be associated with its characteristic phenylethanoids and flavonoids. The paper displays how a combination of phytochemical and biological activity data obtained for extracts of several different species of the same genus may improve the understanding of their potential health benefits and facilitate the identification of compounds that are of possible interest for future biological activity studies. Future studies could focus more on the biological activities of major metabolites found in these species and elucidation of their underlying molecular mechanisms.

## Figures and Tables

**Table 1:** Phytochemical content of methanolic leaf extracts of *G. alypum*, *G. punctata*, *G. cordifolia* and *G. meridionalis* obtained by ultrasound-assisted extraction (mean values ± SD, *n* = 3).

Constituents *	*G. alypum*	*G. punctata*	*G. cordifolia*	*G. meridionalis*
**Antidiabetic Potential**				
Total phenolics ^a^	130.46 ± 5.99 ^A^	98.50 ± 1.23 ^C^	111.35 ± 3.30 ^B^	123.44 ± 0.77 ^A^
Flavonoids ^b^	30.43 ± 0.29 ^C^	48.49 ± 2.37 ^A^	36.54 ± 1.45 ^B^	39.72 ± 0.60 ^B^
Iridoids ^c^	27.49 ± 3.08 ^D^	343.33 ± 4.88 ^A^	311.23 ± 6.20 ^B^	247.37 ± 2.70 ^C^
Condensed tannins ^d^	3.00 ± 0.06 ^D^	4.07 ± 0.11 ^C^	10.02 ± 0.28 ^A^	6.21 ± 0.11 ^B^
**Anticancer Potential**				
Total phenolics ^a^	131.39 ± 2.89 ^B^	152.69 ± 4.87 ^A^	157.31 ± 4.29 ^A^	159.82 ± 2.34 ^A^
Flavonoids ^b^	32.26 ± 1.37 ^C^	63.03 ± 0.72 ^A^	42.23 ± 0.67 ^B^	43.57 ± 0.50 ^B^
Iridoids ^c^	12.07 ± 0.18 ^D^	440.04 ± 8.73 ^A^	290.82 ± 7.49 ^C^	310.25 ± 4.30 ^B^
Condensed tannins ^d^	2.66 ± 0.09 ^C^	7.71 ± 0.25 ^B^	8.76 ± 0.08 ^A^	8.88 ± 0.27 ^A^

* Content expressed as: ^a^ mg gallic acid equivalents (GAE)/g dry extract (DE); ^b^ mg quercetin equivalents (QE)/g DE; ^c^ mg aucubin equivalents (AE)/g DE; ^d^ mg catechin equivalents (CE)/g DE; different capital letters indicate significant differences between variables (*p* < 0.05): A > B > C > D.

**Table 2:** Phytochemical content of methanolic aerial parts extracts of *G. alypum*, *G. punctata*, *G. cordifolia* and *G. meridionalis* obtained by Soxhlet extraction (mean values ± SD, *n* = 3).

Constituents *	*G. alypum*	*G. punctata*	*G. cordifolia*	*G. meridionalis*
Total phenolics ^a^	112.34 ± 2.17 ^A^	79.92 ± 2.18 ^C^	95.59 ± 1.62 ^B^	98.90 ± 3.93 ^B^
Flavonoids ^b^	26.85 ± 0.46 ^B^	43.25 ± 0.31 ^A^	26.19 ± 0.40 ^BC^	25.42 ± 0.24 ^C^

* Content expressed as: ^a^ mg GAE/g DE; ^b^ mg QE/g DE; different capital letters indicate significant differences between variables (*p* < 0.05): A > B > C.

**Table 3:** LC-MS profile of methanolic leaf extracts from *G. alypum* (*GA*), *G. punctata* (*GP*), *G. cordifolia* (*GC*) and *G. meridionalis* (*GM*) obtained by ultrasound-assisted extraction.

Peak	t_R_ (min)	*m/z* (Major Ion in Full MS Indicated in Bold)	Compound Identification (Compound Class Abbreviation)	*GA*	*GP*	*GC*	*GM*
1	1.2	217 [M + ^35^Cl]^−^, 219 [M + ^37^Cl]^−^	Mannitol (O) ^a^	+	+	+	+
2	1.2	377 [M + ^35^Cl]^−^, 379 [M + ^37^Cl]^−^	Sucrose (O) ^a^	+	+	+	+
3	1.2	191 [M–H]^−^	Quinic acid (O) ^a^	–	+	+	+
4	1.7	407 [M–H + HCOOH]^−^	Catalpol (I) ^a^	+	+	–	–
5	2.2	389 [M–H]^−^, 779 [2M–H]^−^	Scandoside (I) ^a^	–	+	–	–
6	2.5	391 [M–H + HCOOH]^−^	Aucubin (I) ^a^	+	+	+	+
7	2.5	407 [M–H + HCOOH]^−^	Monomelittoside (5-Hydroxyaucubin) (I) ^a^	–	–	+	+
8	2.6	371 [M–H]^−^, 417 [M–H + HCOOH]^−^	Deacetylasperuloside (I) ^a^	–	+	+	+
9	4.1	315 [M–H]^−^	1′-*O*-Hydroxytyrosol glucoside (P) ^b^	+ *	–	–	–
10	6.1	341 [M–H]^−^	Caffeoylglucoside isomer (O) ^a^	+	+	–	–
11	6.2	373 [M–H]^−^, 419 [M–H + HCOOH]^−^	Gardoside (I) ^a^	–	+	–	–
12	6.9	505 [M–H]^−^	Hebitol II (6′-*O*-Caffeoyl-β-d-glucopyranosyl-(1→6)-mannitol) (O) ^a^	+	+	+	+
13	7.4	431 [M–H]^−^, 863 [2M–H]^−^	Asperulosidic acid (I) ^a^	–	+	+	+
14	9.4	459 [M–H + HCOOH]^−^	Asperuloside (I) ^a^	–	+	+	+
15	11.2	519 [M–H]^−^, 565 [M–H + HCOOH]^−^	Globularitol (6′-*O*-Feruloyl-β-d-glucopyranosyl-(1→6)-mannitol) (O) ^a^	+	–	–	–
16	11.3	523 [M–H]^−^	Verminoside (6-*O*-Caffeoylcatalpol) (I) ^a^	+	–	–	–
17	11.6	433 [M–H + HCOOH]^−^	Geniposide (I) ^b^	+ *	–	–	–
18	13.1	593 [M–H]^−^	Vicenin-2 (Apigenin-6,8-di-*C*-glucoside) (F) ^a^	+	–	–	–
19	14.4	507 [M–H]^–^, 553 [M–H + HCOOH]^−^	Specioside (6-*O*-(*p*-Coumaroyl)-catalpol) (I) ^a^	+	–	–	–
20	14.8	625 [M–H]^−^	6-Hydroxyluteolin 7-*O*-sophoroside (F) ^a^	+	+	–	–
21	15.4	701 [M–H + HCOOH]^−^	6′-*O*-Benzoyldeacetylasperulosidic acid glucoside (I) ^b^	–	–	+ *	+ *
22	16.1	463 [M–H]^−^, 927 [2M–H]^−^	6-Hydroxyluteolin 7-*O*-glucoside (F) ^a^	+	+	+	+
23	16.7	415 [M–H]^−^, 461 [M–H + HCOOH]^−^	Alpinoside (I) ^a^	+	+	+	+
24	17.1	555 [M–H + HCOOH]^−^	Globularinin (I) ^a^	+	+	–	–
25	18.6	493 [M–H]^−^, 987 [2M–H]^−^	6′-*O*-Benzoyldeacetylasperulosidic acid (I) ^b^	–	+ *	+ *	+ *
26	18.6	555 [M–H + HCOOH]^−^	Globularimin (I) ^a^	+	+	–	–
27	18.6	787 [M–H + HCOOH]^−^	Liriodendrin ((+)-Syringaresinol di-*O*-β-glucopyranoside) (L) ^a^	+	–	–	–
28	18.7	463 [M–H]^−^	Isoquercitrin (Quercetin 3-*O*-glucoside) (F) ^a^	+	+	–	–
29	19.1	511 [M–H + HCOOH]^−^	6′-*O*-Benzoylmonomelittoside (5-Hydroxydumuloside) (I) ^a^	–	–	+	+
30	19.5	477 [M–H]^−^	Calceolarioside A (Desrhamnosyl verbascoside) (P) ^a^	+	–	–	–
31	19.8	787 [M–H]^−^, 1575 [2M–H]^−^	6-Hydroxyluteolin 7-*O*-(6′′′-*O*-caffeoyl)-sophoroside (F) ^a^	–	+	–	–
32	20.2	495 [M–H + HCOOH]^−^	6-*O*-Benzoylaucubin (I) ^a^	–	–	+	+
33	20.6	477 [M–H]^−^	Calceolarioside B (Desrhamnosyl isoverbascoside) (P) ^a^	+	–	–	–
34	21.2	477 [M–H]^−^	Nepetin 7-*O*-glucoside (6-Methoxyluteolin 7-*O*-glucoside) (F) ^b^	+	+ *	+ *	+ *
35	21.2	785 [M–H]^−^	Rossicaside A (P) ^a^	+	+	+	+
36	21.2	623 [M–H]^−^, 1247 [2M–H]^−^	Verbascoside (Acteoside) (P) ^a^	+	+	+	+
37	21.8	653 [M–H]^−^, 1307 [2M–H]^−^	Methoxyverbascoside isomer (P) ^b^	–	–	+ *	+ *
38	22.1	443 [M–2H]^2–^, 887 [M–H]^−^	Trichosanthoside B (P) ^a^	–	+	–	–
39	22.5	539 [M–H + HCOOH]^−^	Globularidin (I) ^a^	+	+	–	–
40	22.8	623 [M–H]^−^	Isoverbascoside (Isoacteoside) (P) ^a^	+	+	+	+
41	22.9	755 [M–H]^−^, 1511 [2M–H]^−^	Trichosanthoside A (P) ^a^	–	+	–	–
42	23.1	771 [M–H]^−^	6-Hydroxyluteolin 7-*O*-(6′′′-*O*-(*p*-coumaroyl))-sophoroside (F) ^a^	–	+	–	–
43	23.5	511 [M–H + HCOOH]^−^	Globularifolin (10-*O*-Benzoylmonomelittoside) (I)^a^	–	–	+	+
44	23.7	623 [M–H]^−^	Forsythoside A (P) ^a^	+	+	+	+
45	24.3	537 [M–H + HCOOH]^−^	Globularin (10-*O*-*trans*-Cinnamoylcatalpol) (I) ^a^	+	+	–	–
46	25.2	477 [M–H]^−^, 523 [M–H + HCOOH]^−^	6′-*O*-Benzoyldeacetylalpinoside (I) ^a^	–	–	+	+
47	25.3	755 [M–H]^−^	Arenarioside (P) ^a^	–	+	–	–
48	25.7	637 [M–H]^−^, 683 [M–H + HCOOH]^−^	Leucosceptoside A (P) ^a^	+	+	+	+
49	26.1	517 [M–H]^−^, 563 [M–H + HCOOH]^−^	10-*O*-(*p*-Coumaroyl)-deacetylasperuloside (I) ^b^	–	+ *	+ *	+ *
50	26.1	491 [M–H]^−^, 983 [2M–H]^−^	6′-*O*-Feruloyl-1′-*O*-hydroxytyrosol glucoside (P) ^b^	+ *	–	–	–
51	28.2	521 [M–H + HCOOH]^−^	Besperuloside (10-*O*-Benzoyldeacetylasperuloside) (I) ^a^	–	+	–	–
52	29.2	573 [M–H + HCOOH]^−^	Globularioside (I) ^a^	+	+	–	–
53	30.4	653 [M–H]^−^, 835 [M–H + mannitol]^−^	Demethoxycentaureidin 6,4′-dimethyl ether (F) ^a^	–	–	+	+
54	32.4	813 [M–H]^−^	Alpinoside-alpinoside dimer (I) ^a^	+	–	–	–
55	33.2	961 [M–H]^−^	Globusintenoside isomer (P) ^a^	+	+	+	+
56	34.1	639 [M–H]^−^	Desrhamnosyl 6′-*O*-caffeoylverbascoside (P) ^b^	+ *	–	–	–
57	34.3	785 [M–H]^−^	6′-*O*-Caffeoylverbascoside (P) ^a^	+	–	–	–
58	35.5	889 [M–H]^−^	Benzoylrossicaside A isomer (P) ^b^	–	+ *	+ *	+ *
59	36.5	935 [M–H + HCOOH]^−^	Globuloside A (Alpinoside-globularin dimer) (I) ^a^	+	–	–	–
60	39.1	799 [M–H]^−^	Galypumoside B (6′-*O*-Feruloylverbascoside) (P) ^a^	+	–	–	–
61	39.3	653 [M–H]^−^, 689 [M + ^35^Cl]^−^, 691 [M + ^37^Cl]^−^	Desrhamnosyl galypumoside B (P) ^b^	+ *	–	–	–
62	40.7	327 [M–H]^−^	Oxo-dihydroxy-octadecenoic acid (O) ^a^	+	+	+	+
63	41.8	789 [M–H]^−^	Galypumoside C (6′-*O*-Menthiafoloylverbascoside) (P) ^b^	+	–	–	–
64	43.4	329 [M–H]^−^	Trihydroxy-octadecenoic acid (O) ^a^	+	+	+	+

^a^ For more information see previously published paper [22]; ^b^ for more information see Table S1; * compounds reported for the first time for a particular *Globularia* species; F: flavonoid; I: iridoid; L: lignan; O: other; P: phenylethanoid.

**Table 4:** LC-MS profile of methanolic aerial parts extracts from *G. alypum* (*GA*), *G. punctata* (*GP*), *G. cordifolia* (*GC*) and *G. meridionalis* (*GM*) obtained by Soxhlet extraction.

Peak	t_R_ (min)	*m/z* (Major Ion in Full MS Indicated in Bold)	Compound Identification (Compound Class Abbreviation)	*GA*	*GP*	*GC*	*GM*
1′	1.2	217 [M + ^35^Cl]^–^, 219 [M + ^37^Cl]^–^	Mannitol (O) ^a^	+	+	+	+
2′	1.2	377 [M + ^35^Cl]^–^, 379 [M + ^37^Cl]^–^	Sucrose (O) ^a^	+	+	+	+
3′	1.7	407 [M–H + HCOOH]^–^	Catalpol (I) ^a^	+	+	–	–
4′	2.1	389 [M–H]^–^, 779 [2M–H]^–^	Scandoside (I) ^a^	–	+	–	–
5′	2.2	407 [M–H + HCOOH]^–^	Monomelittoside (5-Hydroxyaucubin) (I)^a^	–	–	+	+
6′	2.4	371 [M–H]^–^, 417 [M–H + HCOOH]^–^	Deacetylasperuloside (I) ^a^	–	+	+	+
7′	5.9	341 [M–H]^–^	Caffeoylglucoside isomer (O) ^a^	+	+	+	+
8′	5.9	373 [M–H]^–^, 419 [M–H + HCOOH]^–^	Gardoside (I) ^a^	+	+	+	+
9′	6.9	431 [M–H]^–^, 863 [2M–H]^–^	Asperulosidic acid (I) ^a^	–	+	+	+
10′	8.9	459 [M–H + HCOOH]^–^	Asperuloside (I) ^a^	–	+	+	+
11′	10.7	523 [M–H]^–^, 1047 [2M–H]^–^	Verminoside (6-*O*-Caffeoylcatalpol) (I) ^a^	+	–	–	–
12′	10.9	433 [M–H + HCOOH]^–^	Geniposide (I) ^b^	+ *	–	–	–
13′	13.7	507 [M–H]^–^, 553 [M–H + HCOOH]^–^	Specioside (6-*O*-(*p*-Coumaroyl)-catalpol) (I) ^a^	+	–	–	–
14′	14.1	625 [M–H]^–^, 671 [M–H + HCOOH]^–^, 1251 [2M–H]^–^	6-Hydroxyluteolin 7-*O*-sophoroside (F) ^a^	+	+	–	–
15′	14.4	701 [M–H + HCOOH]^–^	6′-*O*-Benzoyldeacetylasperulosidic acid glucoside (I) ^b^	–	–	+ *	+ *
16′	15.2	463 [M–H]^–^, 927 [2M–H]^–^	6-Hydroxyluteolin 7-*O*-glucoside (F) ^a^	+	+	+	+
17′	15.9	415 [M–H]^–^, 461 [M–H + HCOOH]^–^	Alpinoside (I) ^a^	+	+	+	+
18′	16.4	555 [M–H + HCOOH]^–^	Globularinin (I) ^a^	+	+	–	–
19′	17.6	493 [M–H]^–^, 987 [2M–H]^–^	6′-*O*-Benzoyldeacetylasperulosidic acid (I) ^b^	–	+ *	+ *	+ *
20′	17.8	555 [M–H + HCOOH]^–^	Globularimin (I) ^a^	+	+	–	–
21′	17.9	463 [M–H + HCOOH–324]^–^, 787 [M–H + HCOOH]^–^	Liriodendrin ((+)-Syringaresinol di-*O*-β-glucopyranoside) (L) ^a^	+	–	–	–
22′	18.1	511 [M–H + HCOOH]^–^	6′-*O*-Benzoylmonomelittoside (5-Hydroxydumuloside) (I)^a^	–	–	+	+
23′	18.8	477 [M–H]^–^	Calceolarioside A (Desrhamnosyl verbascoside) (P) ^a^	+	–	–	–
24′	19.1	787 [M–H]^–^, 1575 [2M–H]^–^	6-Hydroxyluteolin 7-*O*-(6′′′-*O*-caffeoyl)-sophoroside (F) ^a^	–	+	–	–
25′	19.8	477 [M–H]^–^	Calceolarioside B (Desrhamnosyl isoverbascoside) (P) ^a^	+	–	–	–
26′	20.2	785 [M–H]^–^	Rossicaside A (P) ^a^	+	+	+	+
27′	20.4	623 [M–H]^–^, 1247 [2M–H]^–^	Verbascoside (Acteoside) (P) ^a^	+	+	+	+
28′	20.7	653 [M–H]^–^, 1307 [2M–H]^–^	Methoxyverbascoside isomer (P) ^b^	–	–	+ *	+ *
29′	21.3	443 [M–2H]^2–^, 887 [M–H]^–^	Trichosanthoside B (P) ^a^	–	+	–	–
30′	22.0	623 [M–H]^–^	Isoverbascoside (Isoacteoside) (P) ^a^	+	+	+	+
31′	22.1	755 [M–H]^–^, 1511 [2M–H]^–^	Trichosanthoside A (P) ^a^	–	+	–	–
32′	22.4	771 [M–H]^–^	6-Hydroxyluteolin 7-*O*-(6′′′-*O*-(*p*-coumaroyl)-sophoroside (F)^a^	–	+	–	–
33′	22.5	511 [M–H + HCOOH]^–^	Globularifolin (10-*O*-Benzoylmonomelittoside) (I) ^a^	–	–	+	+
34′	23.0	623 [M–H]^–^	Forsythoside A (P) ^a^	+	+	+	+
35′	23.6	537 [M–H + HCOOH]^–^	Globularin (10-*O*-*trans*-Cinnamoylcatalpol) (I) ^a^	+	+	–	–
36′	24.2	477 [M–H]^–^, 523 [M–H + HCOOH]^–^	6′-*O*-Benzoyldeacetylalpinoside (I) ^a^	–	–	+	+
37′	24.6	755 [M–H]^–^	Arenarioside (P) ^a^	–	+	–	–
38′	24.9	637 [M–H]^–^, 683 [M–H + HCOOH]^–^	Leucosceptoside A (P) ^a^	+	+	+	+
39′	25.1	517 [M–H]^–^, 563 [M–H + HCOOH]^–^	10-*O*-(*p*-Coumaroyl)-deacetylasperuloside (I) ^b^	–	+ *	+ *	+ *
40′	25.2	491 [M–H]^–^, 983 [2M–H]^–^	6′-*O*-Feruloyl-1′-*O*-hydroxytyrosol glucoside (P) ^b^	+ *	–	–	–
41′	27.4	521 [M–H + HCOOH]^–^	Besperuloside (10-*O*-Benzoyldeacetylasperuloside) (I) ^a^	–	+	–	–
42′	28.3	573 [M–H + HCOOH]^–^	Globularioside (I) ^a^	+	+	–	–
43′	29.4	653 [M–H]^–^, 835 [M–H + mannitol]^–^, 1307 [2M–H]^–^	Demethoxycentaureidin 6,4′-dimethyl ether (F) ^a^	–	–	+	+
44′	32.4	961 [M–H]^–^	Globusintenoside isomer (P) ^a^	+	+	+	+
45′	33.5	785 [M–H]^–^	6′-*O*-Caffeoylverbascoside (P) ^a^	+	–	–	–
46′	34.2	269 [M–H]^–^	Apigenin (F) ^a^	+	+	+	+
47′	34.6	889 [M–H]^–^	Benzoylrossicaside A isomer (P) ^b^	–	+ *	+ *	+ *
48′	35.6	935 [M–H + HCOOH]^–^	Globuloside A (Alpinoside-globularin dimer) (I) ^a^	+	–	–	–
49′	38.2	799 [M–H]^–^	Galypumoside B (6′-*O*-Feruloylverbascoside) (P) ^a^	+	–	–	–

^a^ For more information see previously published paper [22]; ^b^ for more information see Table S1; * compounds reported for the first time for a particular *Globularia* species; F: flavonoid; I: iridoid; L: lignan; O: other; P: phenylethanoid.

**Table 5:** DPPH radical scavenging activity of *G. alypum*, *G. punctata*, *G. cordifolia* and *G. meridionalis* methanolic aerial parts extracts expressed as IC_50_ value (μg/mL).

*G. alypum*	*G. punctata*	*G. cordifolia*	*G. meridionalis*	Gallic Acid	Trolox
17.25	24.19	22.68	20.41	0.64	2.71

IC_50_—concentration required to reduce the DPPH radical absorbance by 50%.

**Table 6:** COX-1 inhibitory activity (%) of *G. alypum, G. punctata, G. cordifolia* and *G. meridionalis* (*GM*) aerial parts extracts (mean values ± SD; *n* = 3 for TMPD assay; *n* = 4 for PGE_2_ assay; c = 50 μg/mL).

Assay	*G. alypum*	*G. punctata*	*G. cordifolia*	*G. meridionalis*	Indomethacin
TMPD	51.3 ± 17.4 ^A^	39.9 ± 9.6 ^AB^	37.8 ± 6.6 ^AB^	17.6 ± 7.9 ^B^	2.90 *
PGE_2_	40.6 ± 4.8 ^A^	32.9 ± 2.6 ^A^	26.5 ± 4.7 ^A^	25.7 ± 3.3 ^AB^	1.03 *

TMPD—*N*,*N*,*N*′,*N*′-tetramethyl-*p*-phenylenediamine dihydrochloride; PGE_2_—prostaglandin E_2_; different capital letters indicate significant differences between variables (*p* < 0.05): A > B; * IC_50_ of indomethacin, expressed in μM.

**Table 7:** Growth inhibition zones (mm) of *G. alypum*, *G. punctata*, *G. cordifolia* and *G. meridionalis* methanolic aerial parts extracts against tested bacterial strains (mean values ± S.D., *n* = 3).

Bacterial Strains	*G. alypum*	*G. punctata*	*G. cordifolia*	*G. meridionalis*	Gentamicin ^a^	Norfloxacin ^b^
**Gram-Positive**						
*Bacillus cereus* ATCC 11778	8.3 ± 0.6	9.3 ± 1.2	9.7 ± 0.6	8.0 ± 0.0	22.3 ± 0.6	26.6 ± 0.9
*Bacillus subtilis* ATCC 6633	7.7 ± 0.6	n.d.	8.3 ± 1.5	n.d.	24.7 ± 0.6	41.0 ± 0.0
*Enterococcus faecalis* ATCC 29212	n.d.	13.0 ± 0.0	n.d.	n.d.	15.0 ± 0.0	23.8 ± 0.8
*Staphylococcus aureus* ATCC 6538	25.0 ± 0.0	21.3 ± 0.6	23.0 ± 0.0	23.7 ± 1.5	24.0 ± 0.0	33.8 ± 0.8
**Gram-Negative**						
*Escherichia coli* ATCC 10536	n.d.	24.0 ± 1.7	n.d.	n.d.	23.3 ± 0.6	44.7 ± 1.0
*Klebsiella pneumoniae* MFBF 10402	n.d.	n.d.	n.d.	n.d.	21.0 ± 0.0	7.3 ± 0.5
*Pseudomonas aeruginosa* ATCC 27853	9.7 ± 0.6	n.d.	n.d.	n.d.	24.3 ± 0.6	30.7 ± 2.8

^a^ Positive control, broad-spectrum aminoglycoside antibiotic (c = 0.2 mg/mL, *n* = 3); ^b^ positive control, broad-spectrum fluoroquinolone antibiotic (c = 0.2 mg/mL, *n* = 6); n.d.—growth inhibition not detected with the applied concentration of extract (c = 50 mg/mL).

**Table 8:** Minimum inhibitory concentrations (MICs) and minimum bactericidal concentrations (MBCs) (mg/mL) of *G. alypum*, *G. punctata*, *G. cordifolia* and *G. meridionalis* methanolic aerial parts extracts against tested bacterial strains (mean values, *n* = 3).

Bacterial Strains	*G. alypum*	*G. punctata*	*G. cordifolia*	*G. meridionalis*	Gentamicin ^a^	Verbascoside ^b^
**Gram-Positive**						
*Bacillus cereus* ATCC 11778	11.36 ^c^ 11.36 ^d^	2.84 ^c^ 2.84 ^d^	11.36 ^c^ 11.36 ^d^	11.36 ^c^ n.d. ^d^	0.0015 ^c^ 0.0015 ^d^	n.d. ^c^ n.d. ^d^
*Bacillus subtilis* ATCC 6633	n.d. ^c^ n.d. ^d^	1.89 ^c^ 2.84 ^d^	n.d. ^c^ n.d. ^d^	n.d. ^c^ n.d. ^d^	< 0.0001 ^c^ 0.0002 ^d^	n.d. ^c^ n.d. ^d^
*Enterococcus faecalis* ATCC 29212	n.d. ^c^ * n.d. ^d^	n.d. ^c^ * 5.68 ^d^	n.d. ^c^ * n.d. ^d^	n.d. ^c^ * n.d. ^d^	n.d. ^c^ * 0.0046 ^d^	n.d. ^c^ * n.d. ^d^
**Methicillin-Susceptible *Staphylococcus aureus* (MSSA)**						
*Staphylococcus aureus* ATCC 6538	1.42 ^c^ 1.89 ^d^	1.42 ^c^ 1.42 ^d^	2.84 ^c^ 3.79 ^d^	2.84 ^c^ 2.84 ^d^	0.0006 ^c^ 0.0008 ^d^	0.2272 ^c^ n.d. ^d^
*Staphylococcus aureus* ATCC 29213	4.73 ^c^ 4.73 ^d^	11.36 ^c^ 11.36 ^d^	7.58 ^c^ 11.36 ^d^	11.36 ^c^ 11.36 ^d^	0.0003 ^c^ 0.0007 ^d^	0.2272 ^c^ 0.2272 ^c^
*Staphylococcus aureus* MFBF 505	2.37 ^c^ 2.37 ^d^	1.42 ^c^ 1.42 ^d^	2.84 ^c^ 2.84 ^d^	2.84 ^c^ 2.84 ^d^	n.m. n.m.	n.m. n.m.
*Staphylococcus aureus* MFBF 10661	1.42 ^c^ 1.42 ^d^	1.42 ^c^ 1.42 ^d^	1.89 ^c^ 2.37 ^d^	1.42 ^c^ 1.89 ^d^	n.m. n.m.	n.m. n.m.
*Staphylococcus aureus* MFBF 10666	1.42 ^c^ 1.42 ^d^	1.89 ^c^ 1.89 ^d^	1.89 ^c^ 1.89 ^d^	1.42 ^c^ 1.42 ^d^	n.m. n.m.	n.m. n.m.
**Methicillin-Resistant *Staphylococcus aureus* (MRSA)**						
MRSA MFBF 101	1.89 ^c^ 2.84 ^d^	1.42 ^c^ 1.42 ^d^	2.84 ^c^ 2.84 ^d^	2.84 ^c^ 2.84 ^d^	n.m. n.m.	n.m. n.m.
MRSA MFBF 124	2.84 ^c^ 2.84 ^d^	2.84 ^c^ 2.84 ^d^	3.31 ^c^ 3.79 ^d^	2.84 ^c^ 2.84 ^d^	n.m. n.m.	n.m. n.m.
MRSA MFBF 154	2.84 ^c^ 2.84 ^d^	1.89 ^c^ 2.37 ^d^	2.84 ^c^ 2.84 ^d^	2.84 ^c^ 2.84 ^d^	n.m. n.m.	n.m. n.m.
MRSA MFBF 164	2.84 ^c^ 2.84 ^d^	1.42 ^c^ 1.89 ^d^	2.84 ^c^ 2.84 ^d^	3.79 ^c^ 3.79 ^d^	n.m. n.m.	n.m. n.m.
MRSA MFBF 177	2.84 ^c^ 2.84 ^d^	2.37 ^c^ 2.84 ^d^	3.79 ^c^ 3.79 ^d^	2.84 ^c^ 2.84 ^d^	n.m. n.m.	n.m. n.m.
**Gram-Negative**						
*Escherichia coli* ATCC 10536	n.d. ^c^ n.d. ^d^	1.42 ^c^ 2.84 ^d^	n.d. ^c^ n.d. ^d^	n.d. ^c^ n.d. ^d^	0.0001 ^c^ 0.0015 ^d^	0.2272 ^c^ n.d. ^d^
*Klebsiella pneumoniae* MFBF 10402	n.d. ^c^ n.d. ^d^	n.d. ^c^ n.d. ^d^	n.d. ^c^ n.d. ^d^	n.d. ^c^ n.d. ^d^	0.0003 ^c^ 0.0008 ^d^	n.d. ^c^ n.d. ^d^
*Pseudomonas aeruginosa* ATCC 27853	22.73 ^c^ 22.73 ^d^	n.d. ^c^ n.d. ^d^	n.d. ^c^ n.d. ^d^	n.d. ^c^ n.d. ^d^	0.0011 ^c^ 0.0030 ^d^	0.2272 ^c^ n.d. ^d^

^a^ Positive control, broad-spectrum aminoglycoside antibiotic; ^b^ major phenylethanoid of all investigated species; ^c^ minimum inhibitory concentrations (MICs) evaluated based on the serial broth microdilution method after detection with 1% (*w/v*) TTC; ^d^ minimum bactericidal concentrations (MBCs) evaluated after agar sub-cultivation; n.d.—growth inhibition not detected with tested concentrations of extract (c = 0.36–22.73 mg/mL for *P. aeruginosa*, c = 0.18–11.36 mg/mL for other bacterial species); * color change with TTC was not observed; n.m.—not measured.

**Table 9:** Effect of *G. alypum*, *G. punctata*, *G. cordifolia* and *G. meridionalis* methanolic leaf extracts on viability of A1235 cancer cell line expressed as IC_50_ values (μg/mL).

*G. alypum*	*G. punctata*	*G. cordifolia*	*G. meridionalis*
231.43	140.54	180.42	129.40

IC_50_—concentration required to reduce A1235 cell viability by 50%.

## Data Availability

Data is contained within the article and Supplementary Material.

## Supplementary Materials

The following are available online at https://www.mdpi.com/article/10.3390/ph15050506/s1, Table S1: Chromatographic and spectral data for new* tentatively identified compounds detected by LC-PDA-ESI-MS^n^ using negative ionization mode in methanolic leaf extracts of investigated *Globularia* spp. obtained by ultrasound-assisted extraction; Table S2: Pearson’s correlation coefficients (*r*) for correlations between measured parameters for evaluation of antidiabetic, antioxidant, anti-inflammatory, antimicrobial and anticancer potential, and established contents of secondary metabolites of investigated *Globularia* species; Table S3: Collection data for investigated samples of *G. alypum*, *G. punctata*, *G. cordifolia* and *G. meridionalis* from Croatia; Figure S1: TLC chromatogram of Soxhlet extracts of aerial parts from *G. alypum* (GA), *G. cordifolia* (GC), *G. meridionalis* (GM) and *G. punctata* (GP) and verbascoside reference standard obtained after treatment with 0.05% (*w/v*) methanolic 2,2-diphenyl-1-picrylhydrazyl (DPPH), observed under white light. Stationary phase: silica gel 60 F_254_; mobile phase: ethyl acetate-formic acid-glacial acetic acid-wate*r =* 100:11:11:26 (*v/v/v/v*); Figure S2: TLC chromatogram of Soxhlet extracts of aerial parts from *G. alypum* (GA), *G. cordifolia* (GC), *G. meridionalis* (GM) and *G. punctata* (GP) and verbascoside reference standard obtained after treatment with 1% (*w/v*) methanolic diphenylboric acid-β-ethylamino ester, and 5% (*w/v*) ethanolic polyethylene glycol 4000 (NP/PEG reagent), observed under UV 365 nm. Stationary phase: silica gel 60 F_254_; mobile phase: ethyl acetate-formic acid-glacial acetic acid-wate*r =* 100:11:11:26 (*v/v/v/v*).

## References

[1] (2021). World Health Statistics 2021: Monitoring Health for the SDGs, Sustainable Development Goals.

[2] Saeedi P., Salpea P., Karuranga S., Petersohn I., Malanda B., Gregg E.W., Unwin N., Wild S.H., Williams R. (2020). Mortality attributable to diabetes in 20–79 years old adults, 2019 estimates: Results from the International Diabetes Federation Diabetes Atlas, 9th edition. Diabetes Res. Clin. Pract..

[3] Siti H.N., Kamisah Y., Kamsiah J. (2015). The role of oxidative stress, antioxidants and vascular inflammation in cardiovascular disease (a review). Vascul. Pharmacol..

[4] García-Sánchez A., Miranda-Díaz A.G., Cardona-Muñoz E.G. (2020). The role of oxidative stress in physiopathology and pharmacological treatment with pro- and antioxidant properties in chronic diseases. Oxid. Med. Cell. Longev..

[5] Lima J.E.B.F., Moreira N.C.S., Sakamoto-Hojo E.T. (2022). Mechanisms underlying the pathophysiology of type 2 diabetes: From risk factors to oxidative stress, metabolic dysfunction, and hyperglycemia. Mutat. Res. Genet. Toxicol. Environ. Mutagen..

[6] Giacco F., Brownlee M. (2010). Oxidative stress and diabetic complications. Circ. Res..

[7] Neves J.M., Duarte B., Pinto M., Formiga A., Neves J. (2019). Diabetic foot infection: Causative pathogens and empiric antibiotherapy considerations—The experience of a tertiary center. Int. J. Low. Extrem. Wounds.

[8] Hussein R.A., El-Ansarry A.A., Builders P. (2018). Plants Secondary Metabolites: The Key Drivers of the Pharmacological Actions of Medicinal Plants. Herbal Medicine.

[9] Asraoui F., Kounnoun A., El Cadi H., Cacciola F., El Majdoub Y.O., Alibrando F., Mandolfino F., Dugo P., Mondello L., Louajri A. (2021). Phytochemical investigation and antioxidant activity of *Globularia alypum* L.. Molecules.

[10] Bahlil Y., Krouf D., Mellouk Z., Taleb-Dida N., Guenzet A. (2020). Favorable effects of *Globularia alypum* on cardiometabolic markers in high fructose-fed rats. Nutr. Food Sci..

[11] Mohamed T., Souiy Z., Achour L., Hamden K. (2020). Anti-obesity, anti-hyperglycaemic, anti-antipyretic and analgesic activities of *Globularia alypum* extracts. Arch. Physiol. Biochem..

[12] Ghlissi Z., Kallel R., Sila A., Harrabi B., Atheymen R., Zeghal K., Bougatef A., Sahnoun Z. (2016). *Globularia alypum* methanolic extract improves burn wound healing process and inflammation in rats and possesses antibacterial and antioxidant activities. Biomed. Pharmacother..

[13] Katiri A., Barkaoui M., Msanda F., Boubaker H. (2017). Ethnobotanical survey of medicinal plants used for the treatment of diabetes in the Tizi n’ Test Region (Taroudant Province, Morocco). J. Pharmacogn. Nat. Prod..

[14] Telli A., Esnault M.-A., Khelil A.O.E.H. (2016). An ethnopharmacological survey of plants used in traditional diabetes treatment in south-eastern Algeria (Ouargla province). J. Arid. Environ..

[15] El-Ghazouani F., El-Ouahmani N., Teixidor-Toneu I., Yacoubi B., Zekhnini A. (2021). A survey of medicinal plants used in traditional medicine by women and herbalists from the city of Agadir, southwest of Morocco. Eur. J. Integr. Med..

[16] El-Mokasabi F.M., Al-Sanousi M.F., El-Mabrouk R.M. (2018). Taxonomy and ethnobotany of medicinal plants in Eastern Region of Libya. J. Environ. Sci. Toxicol. Food Technol..

[17] Abouri M., El Mousadik A., Msanda F., Boubaker H., Saadi B., Cherifi K. (2012). An ethnobotanical survey of medicinal plants used in the Tata Province, Morocco. Int. J. Med. Plants.

[18] Afifi-Yazar F.U., Kasabri V., Abu-Dahab R. (2011). Medicinal plants from Jordan in the treatment of cancer: Traditional uses vs. in vitro and in vivo evaluations—Part 1. Planta Med..

[19] Leporatti M.L., Ghedira K. (2009). Comparative analysis of medicinal plants used in traditional medicine in Italy and Tunisia. J. Ethnobiol. Ethnomed..

[20] Helmstädter A. (2016). Ethnopharmacology in the work of Melville William Hilton-Simpson (1881–1938)—Historical analysis and current research opportunities. Pharmazie.

[21] Irki S., Mahmoudi Y., Hamidi N. (2019). Histological study and cytotoxic effect of *Globularia alypum* leaves. Algerian J. Nat. Prod..

[22] Friščić M., Bucar F., Hazler Pilepić K. (2016). LC-PDA-ESI-MS^n^ analysis of phenolic and iridoid compounds from *Globularia* spp.. J. Mass Spectrom..

[23] Friščić M., Maslo S., Garić R., Maleš Ž., Hazler Pilepić K. (2018). Comparative analysis of specialized metabolites and antioxidant capacity in vitro of different natural populations of *Globularia* spp.. Acta Bot. Croat..

[24] Sertić M., Crkvenčić M., Mornar A., Hazler Pilepić K., Nigović B., Maleš Ž. (2015). Analysis of aucubin and catalpol content in different plant parts of four *Globularia* species. J. Appl. Bot. Food Qual..

[25] Eibl R., Meier P., Stutz I., Schildberger D., Hühn T., Eibl D. (2018). Plant cell culture technology in the cosmetics and food industries: Current state and future trends. Appl. Microbiol. Biotechnol..

[26] Sipahi H., Becker K., Gostner J.M., Charehsaz M., Kirmizibekmez H., Schennach H., Aydin A., Fuchs D. (2014). Effects of globularifolin on cell survival, nuclear factor-κB activity, neopterin production, tryptophan breakdown and free radicals in vitro. Fitoterapia.

[27] Yu Y., Fu X., Ran Q., Yang K., Wen Y., Li H., Wang F. (2017). Globularifolin exerts anticancer effects on glioma U87 cells through inhibition of Akt/mTOR and MEK/ERK signaling pathways in vitro and inhibits tumor growth in vivo. Biochimie.

[28] Chen Y., Wang Z., Liu M., Wang X., Zhang L., Gong C. (2018). Globularifolin inhibits CAMA-1 human breast cancer cell line via cell cycle arrest, apoptosis and inhibition of PI3K/AKT signalling pathway. Trop. J. Pharm. Res..

[29] Tundis R., Bonesi M., Menichini F., Loizzo M.R., Conforti F., Statti G., Pirisi F.M., Menichini F. (2012). Antioxidant and anti- cholinesterase activity of *Globularia meridionalis* extracts and isolated constituents. Nat. Prod. Commun..

[30] Rodríguez-Pérez C., Zengin G., Segura-Carretero A., Lobine D., Mahomoodally M.F. (2018). Chemical fingerprint and bioactivity evaluation of *Globularia orientalis* L. and *Globularia trichosantha* Fisch. & C. A. Mey. using non-targeted HPLC-ESI-QTOF-MS approach. Phytochem. Anal..

[31] Hazler Pilepić K., Friščić M., Duran A., Maslo S., Garić R., Čuljak S., Šutalo K. (2016). Contribution to *Globularia phylogeny* based on nuclear ribosomal spacer and two chloroplast DNA regions. Period. Biol..

[32] Tutin T.G., Tutin T.G., Heywood V.H., Burges N.A., Moore D.M., Valentine D.H., Walters S.M., Webb D.A. (1972). *Globularia* L.. Flora Europaea.

[33] Amessis-Ouchemoukh N., Abu-Reidah I.M., Quirantes-Piné R., Rodríguez-Pérez C., Madani K., Fernández-Gutiérrez A., Segura-Carretero A. (2014). Tentative characterisation of iridoids, phenylethanoid glycosides and flavonoid derivatives from *Globularia alypum* L. (Globulariaceae) leaves by LC-ESI-QTOF-MS. Phytochem. Anal..

[34] Bouriche H., Kada S., Senator A., Demirtas I., Ozen T., Toptanci B.C., Kizil G., Kizil M. (2017). Phenolic content and biomolecule oxidation protective activity of *Globularia alypum* extracts. Braz. Arch. Biol. Technol..

[35] Es-Safi N.-E., Kollmann A., Khlifi S., Ducrot P.-H. (2007). Antioxidative effect of compounds isolated from *Globularia alypum* L. structure-activity relationship. LWT Food Sci. Technol..

[36] Kirmizibekmez H., Bassarello C., Piacente S., Çaliş İ. (2008). Phenylethyl glycosides from *Globularia alypum* growing in Turkey. Helv. Chim. Acta.

[37] Feriani A., del Mar Contreras M., Talhaoui N., Gómez-Caravaca A.M., Taamalli A., Segura-Carretero A., Ghazouani L., El Feki A., Allagui M.S. (2017). Protective effect of *Globularia alypum* leaves against deltamethrin-induced nephrotoxicity in rats and determination of its bioactive compounds using high-performance liquid chromatography coupled with electrospray ionization tandem quadrupole-time-of-flight mass spectrometry. J. Funct. Foods.

[38] Kirmizibekmez H., Akbay P., Sticher O., Çaliş İ. (2003). Iridoids from *Globularia dumulosa*. Z. Naturforsch. C.

[39] Kirmizibekmez H., Çaliş İ., Piacente S., Pizza C. (2004). Phenolic compounds from *Globularia cordifolia*. Turk. J. Chem..

[40] Kirmizibekmez H., Bassarello C., Piacente S., Akaydin G., Çaliş İ. (2009). Flavonoid, phenylethanoid and iridoid glycosides from *Globularia aphyllanthes*. Z. Naturforsch. B.

[41] Merghache S., Zerriouh M., Merghache D., Tabti B., Djaziri R., Ghalem S. (2013). Evaluation of hypoglycaemic and hypolipidemic activities of globularin isolated from *Globularia alypum* L. in normal and streptozotocin-induced diabetic rats. J. Appl. Pharm. Sci..

[42] Klimek B. (1988). Acylated 6-hydroxyluteolin diglucosides from *Globularia elongata*. Phytochemistry.

[43] Kirmizibekmez H., Çaliş İ., Akbay P., Sticher O. (2003). Iridoid and bisiridoid glycosides from *Globularia cordifolia*. Z. Naturforsch. C.

[44] Ben Hassine B., Bui A., Mighri Z. (1982). Contribution a l’etude des plantes medicinales Tunisiennes. Identification des acides phenols de *Globularia alypum* L. par C.C.M. bidimensionnelle et H.P.L.C. J. Soc. Chim. Tunisie.

[45] Ben Hassine B., Bui A.M., Mighri Z., Cavé A. (1982). Flavonoïdes et anthocyanes de *Globularia alypum* L.. Plantes Méd. Phytothér..

[46] Liu S.-K., Hao H., Bian Y., Ge Y.-X., Lu S., Xie H.-X., Wang K.-M., Tao H., Yuan C., Zhang J. (2021). Discovery of new α-glucosidase inhibitors: Structure-based virtual screening and biological evaluation. Front. Chem..

[47] Ouffai K., Azzi R., Abbou F., Mahdi S., El Haci I.A., Belyagoubi-Benhammou N., Bekkara F.A., Lahfa F.B. (2021). Phenolics compounds, evaluation of Alpha-amylase, alpha-glucosidase inhibitory capacity and antioxidant effect from *Globularia alypum* L.. Vegetos.

[48] Hajji N., Jabri M.-A., Tounsi H., Wanes D., Ben El Hadj Ali I., Boulila A., Marzouki L., Sebai H. (2018). Phytochemical analysis by HPLC-PDA/ESI-MS of *Globularia alypum* aqueous extract and mechanism of its protective effect on experimental colitis induced by acetic acid in rat. J. Funct. Foods.

[49] Liu Q., Hu H.-J., Li P.-F., Yang Y.-B., Wu L.-H., Chou G.-X., Wang Z.-T. (2014). Diterpenoids and phenylethanoid glycosides from the roots of *Clerodendrum bungei* and their inhibitory effects against angiotensin converting enzyme and α-glucosidase. Phytochemistry.

[50] Hadrich F., Bouallagui Z., Junkyu H., Isoda H., Sayadi S. (2015). The α-glucosidase and α-amylase enzyme inhibitory of hydroxytyrosol and oleuropein. J. Oleo Sci..

[51] Adisakwattana S., Chantarasinlapin P., Thammarat H., Yibchok-Anun S. (2009). A series of cinnamic acid derivatives and their inibitory activity on intestinal α-glucosidase. J. Enzyme Inhib. Med. Chem..

[52] Adem Ş., Eyupoglu V., Sarfraz I., Rasul A., Zahoor A.F., Ali M., Abdalla M., Ibrahim I.M., Elfiky A.A. (2021). Caffeic acid derivatives (CAFDs) as inhibitors of SARS-CoV-2: CAFDs-based functional foods as a potential alternative approach to combat COVID-19. Phytomedicine.

[53] Kawabata J., Mizuhata K., Sato E., Nishioka T., Aoyama Y., Kasai T. (2003). 6-Hydroxyflavonoids as α-glucosidase inhibitors from marjoram (*Origanum majorana*) leaves. Biosci. Biotechnol. Biochem..

[54] Islam M.N., Ishita I.J., Jung H.A., Choi J.S. (2014). Vicenin 2 isolated from *Artemisia capillaris* exhibited potent anti-glycation properties. Food Chem. Toxicol..

[55] Mohamed J., Nazratun Nafizah A.H., Zariyantey A.H., Budin S.B. (2016). Mechanisms of diabetes-induced liver damage: The role of oxidative stress and inflammation. Sultan Qaboos Univ. Med. J..

[56] Zennaki S., Krouf D., Taleb-Senouci D., Bouchenak M. (2009). *Globularia alypum* L. lyophilized methanolic extract decreases hyperglycemia and improves antioxidant status in various tissues of streptozotocin-induced diabetic rats. J. Compl. Integr. Med..

[57] Khlifi D., Hamdi M., El Hayouni A., Cazaux S., Souchard J.P., Couderc F., Bouajila J. (2011). Global chemical composition and antioxidant and anti-tuberculosis activities of various extracts of *Globularia alypum* L. (Globulariaceae) leaves. Molecules.

[58] Taleb-Dida N., Krouf D., Bouchenak M. (2011). *Globularia alypum* aqueous extract decreases hypertriglyceridemia and ameliorates oxidative status of the muscle, kidney, and heart in rats fed a high-fructose diet. Nutr. Res..

[59] Djellouli F., Kaddour A., Krouf D. (2021). The antioxidant and anti-inflammatory effect of *Globularia alypum* aqueous extract in hypercholesterolemic rats. South Asian J. Exp. Biol..

[60] Fournial A., Grizaud C.-M., Mondon P., Le Moigne C. (2020). Extract of Plant Origin of *Globularia* and Method for Obtaining Said Extract by In Vitro Plant Culture. US Patent.

[61] Soldado D., Bessa R.J.B., Jerónimo E. (2021). Condensed tannins as antioxidants in ruminants—Effectiveness and action mechanisms to improve animal antioxidant status and oxidative stability of products. Animals.

[62] Mahbob E.N.M., Ahmad R., Ahmad S. (2014). Nitric oxide (NO) radical inhibitory of *Hedyotis philippinensis* and its marker compound, asperuloside. Mal. J. Fund. Appl. Sci..

[63] Hajji N., Wannes D., Jabri M.-A., Rtibi K., Tounsi H., Abdellaoui A., Sebai H. (2020). Purgative/laxative actions of *Globularia alypum* aqueous extract on gastrointestinal-physiological function and against loperamide-induced constipation coupled to oxidative stress and inflammation in rats. Neurogastroenterol. Motil..

[64] Çaliş İ., Kirmizibekmez H., Rüegger H., Sticher O. (1999). Phenylethanoid glycosides from *Globularia trichosantha*. J. Nat. Prod..

[65] Çaliş İ., Kirmizibekmez H., Taşdemir D., Sticher O., Ireland C.M. (2002). Sugar esters from *Globularia orientalis*. Z. Naturforsch. C.

[66] Hsieh P.-F., Yu C.-C., Chu P.-M., Hsieh P.-L. (2021). Verbascoside protects gingival cells against high glucose-induced oxidative stress via PKC/HMGB1/RAGE/NFκB pathway. Antioxidants.

[67] Es-Safi N.-E., Khlifi S., Kollmann A., Kerhoas L., El Abbouyi A., Ducrot P.H. (2006). Iridoid glucosides from the aerial parts of *Globularia alypum* L. (Globulariaceae). Chem. Pharm. Bull..

[68] Taghzouti O.K., Balouiri M., Ouedrhiri W., Ech chahad A., Romane A. (2016). In vitro evaluation of the antioxidant and antimicrobial effects of *Globularia alypum* L. extracts. J. Mater. Environ. Sci..

[69] Mansour R.B., Wasli H., Serairi-Beji R., Bourgou S., Dakhlaoui S., Selmi S., Khamessi S., Hammami M., Ksouri R., Megdiche-Ksouri W. (2020). In vivo gastroprotective effect and biological potentialities of six Tunisian medicinal plants using multivariate data treatment. Plant Biosyst..

[70] Ahn J.H., Jo Y.H., Kim S.B., Turk A., Oh K.-E., Hwang B.Y., Lee K.Y., Lee M.K. (2018). Identification of antioxidant constituents of the aerial part of *Plantago asiatica* using LC–MS/MS coupled DPPH assay. Phytochem. Lett..

[71] Wagner H., Bladt S. (1996). Plant Drug Analysis: A Thin Layer Chromatography Atlas.

[72] Burgos C., Muñoz-Mingarro D., Navarro I., Martín-Cordero C., Acero N. (2020). Neuroprotective potential of verbascoside isolated from *Acanthus mollis* L. leaves through its enzymatic inhibition and free radical scavenging ability. Antioxidants.

[73] Pannunzio A., Coluccia M. (2018). Cyclooxygenase-1 (COX-1) and COX-1 inhibitors in cancer: A review of oncology and medicinal chemistry literature. Pharmaceuticals.

[74] Yang C., Li P., Ding X., Sui H.C., Rao S., Hsu C.-H., Leung W.-P., Cheng G.-J., Wang P., Zhu B.T. (2020). Mechanism for the reactivation of the peroxidase activity of human cyclooxygenases: Investigation using phenol as a reducing cosubstrate. Sci. Rep..

[75] Amessis-Ouchemoukh N., Madani K., Falé P.L.V., Serralheiro M.L., Araújo M.E.M. (2014). Antioxidant capacity and phenolic contents of some Mediterranean medicinal plants and their potential role in the inhibition of cyclooxygenase-1 and acetylcholinesterase activities. Ind. Crop. Prod..

[76] Sahpaz S., Garbacki N., Tits M., Bailleul F. (2002). Isolation and pharmacological activity of phenylpropanoid esters from *Marrubium vulgare*. J. Ethnopharmacol..

[77] Uddin M.J., Ali Reza A.S.M., Abdullah-Al-Mamun M., Kabir M.S.H., Nasrin M.S., Akhter S., Arman M.S.I., Rahman M.A. (2018). Antinociceptive and anxiolytic and sedative effects of methanol extract of *Anisomeles indica*: An experimental assessment in mice and computer aided models. Front. Pharmacol..

[78] Chen Y., Liu Q., Shan Z., Zhao Y., Li M., Wang B., Zheng X., Feng W. (2019). The protective effect and mechanism of catalpol on high glucose-induced podocyte injury. BMC Complement. Alter. Med..

[79] Bhattamisra S.K., Yap K.H., Rao V., Choudhury H. (2020). Multiple biological effects of an iridoid glucoside, catalpol, and its underlying molecular mechanisms. Biomolecules.

[80] He J., Lu X., Wei T., Dong Y., Cai Z., Tang L., Liu M. (2018). Asperuloside and asperulosidic acid exert an anti-inflammatory effect via suppression of the NF-κB and MAPK signaling pathways in LPS-induced RAW 264.7 macrophages. Int. J. Mol. Sci..

[81] Li J., Zheng X., Li X., Yang J., Liu W., Yang L., Liu B. (2022). Study on the protective effect and mechanism of Liriodendrin on radiation enteritis in mice. J. Radiat. Res..

[82] Galli A., Marciani P., Marku A., Ghislanzoni S., Bertuzzi F., Rossi R., Di Giancamillo A., Castagna M., Perego C. (2020). Verbascoside protects pancreatic β-cells against ER-stress. Biomedicines.

[83] Çaliş İ., Kirmizibekmez H., Sticher O. (2001). Iridoid glycosides from *Globularia trichosantha*. J. Nat. Prod..

[84] Kadioğlu S., Kadioğlu B., Karagöz Sezel K. (2021). Ethnobotanical properties of natural plants in Kop Mountain Pass (Bayburt/ Turkey). BioDiCon.

[85] Pessoa e Costa T., Duarte B., João A.L., Coelho M., Formiga A., Pinto M., Neves J. (2020). Multidrug-resistant bacteria in diabetic foot infections: Experience from a portuguese tertiary centre. Int. Wound J..

[86] Macdonald K.E., Boeckh S., Stacey H.J., Jones J.D. (2021). The microbiology of diabetic foot infections: A meta-analysis. BMC Infect. Dis..

[87] Shanab S., Doro B., Auzi A. (2021). Phytochemical screening and antibacterial activity of Libyan *Globularia alypum*. Khalij-Libya J. Dent. Med. Res..

[88] Boussoualim N., Trabsa H., Krache I., Arrar L., Baghiani A. (2014). Anti-bacterial and β-lactamase inhibitory effects of *Anchusa azurea* and *Globularia alypum* extracts. Res. J. Pharm. Biol. Chem. Sci..

[89] Bouabdelli F., Djelloul A., Kaid-Omar Z., Semmoud A., Addou A. (2012). Antimicrobial activity of 22 plants used in urolithiasis medicine in Western Algeria. Asian Pac. J. Trop. Dis..

[90] Kraza L., Mourad S.M., Halis Y. (2020). In vitro investigation of the antioxidant and antimicrobial effects of hydro-alcoholic and aqueous extracts of *Globularia alypum* L.. ASN.

[91] Bogdadi H.A.A., Kokoska L., Havlik J., Kloucek P., Rada V., Vorisek K. (2007). In vitro antimicrobial activity of some Libyan medicinal plant extracts. Pharm. Biol..

[92] Volk R.-B. (2008). A newly developed assay for the quantitative determination of antimicrobial (anticyanobacterial) activity of both hydrophilic and lipophilic test compounds without any restriction. Microbiol. Res..

[93] Akroum S., Bendjeddou D., Satta D., Lalaoui K. (2009). Antibacterial activity and acute toxicity effect of flavonoids extracted from *Mentha longifolia*. Am.-Eur. J. Sci. Res..

[94] Wang M., Firman J., Liu L.S., Yam K. (2019). A review on flavonoid apigenin: Dietary intake, ADME, antimicrobial effects, and interactions with human gut microbiota. Biomed. Res. Int..

[95] Nazemiyeh H., Rahman M.M., Gibbons S., Nahar L., Delazar A., Ghahramani M.-A., Talebpour A.-H., Sarker S.D. (2008). Assessment of the antibacterial activity of phenylethanoid glycosides from *Phlomis lanceolata* against multiple-drug-resistant strains of *Staphylococcus aureus*. J. Nat. Med..

[96] Shikanga E.A., Combrinck S., Regnier T. (2010). South African *Lippia* herbal infusions: Total phenolic content, antioxidant and antibacterial activities. S. Afr. J. Bot..

[97] Radev R. (2010). Pharmacological effects of phenylethanoid glycosides. J. Clin. Med..

[98] Dafni U., Tsourti Z., Alatsathianos I. (2019). Breast cancer statistics in the European Union: Incidence and survival across European countries. Breast Care.

[99] Waks A.G., Winer E.P. (2019). Breast cancer treatment: A review. JAMA.

[100] Holliday D.L., Speirs V. (2011). Choosing the right cell line for breast cancer research. Breast Cancer Res..

[101] Oronsky B., Reid T.R., Oronsky A., Sandhu N., Knox S.J. (2021). A review of newly diagnosed glioblastoma. Front. Oncol..

[102] Yang B., Wang N., Wang S., Li X., Zheng Y., Li M., Song J., Zhang F., Mei W., Lin Y. (2019). Network-pharmacology-based identification of caveolin-1 as a key target of *Oldenlandia diffusa* to suppress breast cancer metastasis. Biomed. Pharmacother..

[103] Artanti N., Hanafi M., Andriyani R., Saraswati V., Udin Z., Lotulung P.D., Fujita K.I., Usuki Y. (2015). Isolation of an anti-cancer asperuloside from *Hedyotis corymbosa* L.. J. Trop. Life Sci..

[104] Mahibalan S., Rao P.C., Khan R., Basha A., Siddareddy R., Masubuti H., Fujimoto Y., Begum A.S. (2016). Cytotoxic constituents of *Oldenlandia umbellata* and isolation of a new symmetrical coumarin dimer. Med. Chem. Res..

[105] Bawadi H.A., Bansode R.R., Trappey II A., Truax R.E., Losso J.N. (2005). Inhibition of Caco-2 colon, MCF-7 and Hs578T breast, and DU 145 prostatic cancer cell proliferation by water-soluble black bean condensed tannins. Cancer Lett..

[106] Şenol H., Tulay P., Ergören M.Ç., Hanoğlu A., Çaliş İ., Mocan G. (2021). Cytotoxic effects of verbascoside on MCF-7 and MDA-MB-231. Turk. J. Pharm. Sci..

[107] Khalaf H.A.A., Jasim R.A., Ibrahim I.T. (2021). Verbascoside—A review of its antitumor activities. Pharmacol. Pharm..

[108] Ma Y.C., Zhang M., Xu W.T., Feng S.X., Lei M., Yi B. (2014). Chemical constituents from *Callicarpa nudiflora* and their cytotoxic activities. China J. Chin. Mater. Med..

[109] Mansour R.B., Gargouri B., Gargouri B., Elloumi N., Jilani I.B.H., Ghrabi-Grammar Z., Lassoued S. (2012). Investigation of antioxidant activity of alcoholic extract of *Globularia alypum* L.. J. Med. Plants Res..

[110] Lee K.-W., Kim H.J., Lee Y.S., Park H.-J., Choi J.-W., Ha J., Lee K.-T. (2007). Acteoside inhibits human promyelocytic HL-60 leukemia cell proliferation via inducing cell cycle arrest at G_0_/G_1_ phase and differentiation into monocyte. Carcinogenesis.

[111] Wu L., Georgiev M.I., Cao H., Nahar L., El-Seedi H.R., Sarker S.D., Xiao J., Lu B. (2020). Therapeutic potential of phenylethanoid glycosides: A systematic review. Med. Res. Rev..

[112] Bljajić K., Petlevski R., Vujić L., Čačić A., Šoštarić N., Jablan J., Saraiva de Carvalho I., Zovko Končić M. (2017). Chemical composition, antioxidant and α-glucosidase-inhibiting activities of the aqueous and hydroethanolic extracts of *Vaccinium myrtillus* leaves. Molecules.

[113] Habig W.H., Jakoby W.B. (1981). Assay for differentiation of glutathione S-transferases. Methods Enzymol..

[114] Ellman G.L. (1958). A colorimetric method for determining low concentrations of mercaptans. Arch. Biochem. Biophys..

[115] Kumar P., Nagarajan A., Uchil P.D. (2018). Analysis of cell viability by the lactate dehydrogenase assay. Cold Spring Harb. Protoc..

[116] Yang U.J., Park T.-S., Shi S.-M. (2013). Protective effect of chlorophyllin and lycopene from water spinach extract on cytotoxicity and oxidative stress induced by heavy metals in human hepatoma cells. J. Toxicol. Environ. Health A.

[117] Wang J., Yue Y.-D., Tang F., Sun J. (2012). TLC screening for antioxidant activity of extracts from fifteen bamboo species and identification of antioxidant flavone glycosides from leaves of *Bambusa textilis* McClure. Molecules.

[118] Fiebich B.L., Grozdeva M., Hess S., Hüll M., Danesch U., Bodensieck A., Bauer R. (2005). *Petasites hybridus* extracts in vitro inhibit COX-2 and PGE_2_ release by direct interaction with the enzyme and by preventing p42/44 MAP kinase activation in rat primary microglial cells. Planta Med..

[119] Reiniger E.A., Bauer R. (2006). Prostaglandin-H-synthase (PGHS)-1 and -2 microtiter assays for the testing of herbal drugs and in vitro inhibition of PGHS-isoenzyms by polyunsaturated fatty acids from *Platycodi radix*. Phytomedicine.

[120] Copeland R.A., Williams J.M., Giannaras J., Nurnberg S., Covington M., Pinto D., Pick S., Trzaskos J.M. (1994). Mechanism of selective inhibition of the inducible isoform of prostaglandin G/H synthase. Proc. Natl. Acad. Sci. USA.

[121] Council of Europe (2006). European Pharmacopoeia.

[122] Clinical and Laboratory Standards Institute (2012). Methods for Dilution Antimicrobial Susceptibility Tests for Bacteria that Grow Aerobically, Approved standard-.

[123] Lee D.D., Lee E.Y., Jeong S.H., Chang C.L. (2007). Evaluation of a colorimetric broth microdilution method for antimicrobial susceptibility testing using 2,3,5-triphenyltetrazolium chloride. Korean J. Clin. Microbiol..

[124] Giard D.J., Aaronson S.A., Todaro G.J., Arnstein P., Kersey J.H., Dosik H., Parks W.P. (1973). In vitro cultivation of human tumors: Establishment of cell lines derived from a series of solid tumors. J. Natl. Cancer Inst..

[125] Madunić J., Matulić M., Friščić M., Hazler Pilepić K. (2016). Evaluation of the cytotoxic activity of *Hypericum* spp. on human glioblastoma A1235 and breast cancer MDA MB-231 cells. J. Environ. Sci. Health Part A.

