# Supplementary material for: Globularia alypum L. and Related Species: LC-MS Profiles and Antidiabetic, Antioxidant, Anti-Inflammatory, Antibacterial and Anticancer Potential
Source: Pharmaceuticals (Basel). 2022 Apr 21;15(5):506. doi: 10.3390/ph15050506 (PMC9146695; doi:10.3390/ph15050506)
Supplement: Supplementary file 1 [file pharmaceuticals-15-00506-s001.zip › pharmaceuticals-1638802-supplementary.pdf]

Table S1. Chromatographic and spectral data for new* tentatively identified compounds detected by LC-PDA-ESI-MS^n using negative ionization mode in methanolic leaf extracts of investigated *Globularia* spp. obtained by ultrasound-assisted extraction.

Peak	Compound Identification (Compound Class Abbreviation)	Mr	t_R (min)	UV λmax (nm)	*m/z* (Major Ion in Full MS Indicated in Bold) and MS^n (MS^2–MS^4) Fragments (% Relative Abundance)
**9**	1′-*O*-Hydroxytyrosol glucoside (P)	316.30	4.1	197, 224, 282	**315** [M−H]$^-$<br>**MS^2:** 315 → 135 (100), 153 (7);<br>**MS^3:** —; **MS^4:** —
**17**	Geniposide (I)	388.37	11.6	193, 241	**433** [M–H + HCOOH]$^-$<br>**MS^2:** 433 → 225 (100), 387 (48);<br>**MS^3:** 225 → 123 (100); **MS^4:** 123 → 105 (100), 123 (48)
**21**	6'-*O*-Benzoyldeacetylasperulosidic acid glucoside (I)	656.59	15.4	195, 238	**701** [M–H + HCOOH]$^-$<br>**MS^2:** 701 → 493 (100), 655 (14); **MS^3:** 493 → 165 (33), 183 (100), 209 (67), 227 (93), 371 (87);<br>**MS^4:** 183 → 99 (16), 137 (42), 139 (50), 153 (31), 165 (100)
**25**	6'-*O*-Benzoyldeacetylasperulosidic acid (I)	494.45	18.6	196, 232	**493** [M−H]$^-$, 987 [2M−H]$^-$<br>**MS^2:** 493 → 165 (31), 183 (77), 209 (59), 227 (71), 371 (100), 447 (57); **MS^3:** 371 → 137 (26), 165 (20), 183 (19), 209 (100); 183 → 137 (44), 139 (48), 153 (35), 165 (100); 227 → 183 (100); **MS^4:** 165 → 121 (21), 135 (100), 137 (44), 147 (42)
**34**	Nepetin 7-*O*-glucoside (F)	478.40	21.2	198, 213, 284, 344	**477** [M−H]$^-$<br>**MS^2:** 477 → 315 (100), 462 (20); **MS^3:** 315 → 300 (100); **MS^4:** 300 → 137 (16), 174 (20), 200 (44), 216 (100), 227 (68), 228 (73), 241 (43), 255 (53), 256 (63), 272 (99), 283 (39), 299 (83), 300 (36)
**37**	Methoxyverbascoside isomer (P)	654.62	21.8	198, 221, 331	**653** [M−H]$^-$, 1307 [2M−H]$^-$<br>**MS^2:** 653 → 461 (100), 315 (46);<br>**MS^3:** 461 → 135 (37), 161 (12), 297 (12), 315 (100); **MS^4:** 315 → 135 (100)
**49**	10-*O*-(*p*-Coumaroyl)-deacetylasperuloside (I)	518.47	26.1	191, 232, 315	**517** [M−H]$^-$, 563 [M–H + HCOOH]$^-$<br>**MS^2:** 517 → 147 (67), 163 (35), 191 (100), 293 (16), 355 (32);<br>**MS^3:** 191 → 147 (100), 163 (37); **MS^4:** 147 → 91 (15), 103 (9), 119 (100), 147 (72)
**50**	6′-*O*-Feruloyl-1′-*O*-hydroxytyrosol glucoside (P)	492.46	26.1	197, 236, 292, 330	**491** [M−H]$^-$, 983 [2M−H]$^-$<br>**MS^2:** 491 → 161 (100), 175 (93), 179 (14), 281 (10), 297 (21), 315 (52), 337 (62);<br>**MS^3:** 161 → 133 (100), 161 (35); **MS^4:** 133 → 133 (100)
**56**	Desrhamnosyl 6′-*O*-caffeoylverbascoside (P)	640.59	34.1	195, 218, 306, 327	**639** [M−H]$^-$<br>**MS^2:** 639 → 477 (100);<br>**MS^3:** 477 → 161 (100), 179 (11), 315 (21); **MS^4:** 161 → 133 (100), 161 (36)
**58**	Benzoylrossicaside A isomer (P)	890.83	35.5	196, 223, 332	**889** [M−H]$^-$<br>**MS^2:** 889 → 605 (35), 727 (84), 767 (100);<br>**MS^3:** 767 → 605 (100); **MS^4:** 605 → 297 (8), 315 (12), 443 (22), 461 (100)
**61**	Desrhamnosyl galypumoside B (P)	654.61	39.3	197, 217, 301, 328	**653** [M−H]$^-$, 689 [M + ^{35}Cl]$^-$, 691 [M + ^{37}Cl]$^-$<br>**MS^2:** 653 → 459 (8), 477 (100), 491 (59);<br>**MS^3:** 477 → 161 (100), 179 (6), 315 (21); **MS^4:** 161 → 133 (100), 161 (43)
**63**	Galypumoside C (P)	790.80	41.8	197, 214, 291, 334	**789** [M−H]$^-$<br>**MS^2:** 789 → 461 (24), 623 (17), 627 (100);<br>**MS^3:** 627 → 461 (100); **MS^4:** 461 → 135 (35), 161 (11), 297 (11), 315 (100)

* Additional to previously published data [22]; F: flavonoid; I: iridoid; P: phenylethanoid.

Table S2. Pearson's correlation coefficients (*r*) for correlations between measured parameters for evaluation of antidiabetic, antioxidant, anti-inflammatory, antimicrobial and anticancer potential, and established contents of secondary metabolites of investigated *Globularia* species.

Investigated Parameters	Total Phenolics	Flavonoids	Iridoids	Condensed Tannins
α-Glucosidase activity	−0.51	0.09	0.65	0.33
Glutathione S-transferase (GST) activity in Hep G2 cells cultured in hyperglycemic conditions	0.32	0.46	**0.73***	**0.94*****
Glutathione peroxidase (GPx) activity in Hep G2 cells cultured in hyperglycemic conditions	0.20	0.17	0.42	**0.79***
Free thiol groups (-SH) content in Hep G2 cells cultured in hyperglycemic conditions	0.28	0.63	0.54	0.21
Reduced glutathione (GSH) content in Hep G2 cells cultured in hyperglycemic conditions	0.45	0.65	0.67	0.70
Viability of human hepatocellular carcinoma (Hep G2) cells cultured in hyperglycemic conditions (LDH assay)	**0.75***	**0.88****	0.44	0.15
Viability of human hepatocellular carcinoma (Hep G2) cells cultured in hyperglycemic conditions (MTT assay)	−0.18	0.13	0.23	0.02
DPPH radical scavenging activity	**−0.96***	0.65	n.m.	n.m.
Cyclooxygenase-1 (COX-1) inhibitory activity (TMPD assay)	0.23	0.22	n.m.	n.m.
Cyclooxygenase-1 (COX-1) inhibitory activity (PGE_2 assay)	0.40	0.20	n.m.	n.m.
Minimum inhibitory concentration (MIC) against *Bacillus cereus* ATCC 11778	0.84	**−0.998****	n.m.	n.m.
Minimum inhibitory concentration (MIC) against *Staphylococcus aureus* ATCC 6538	0.05	−0.62	n.m.	n.m.
Minimum inhibitory concentration (MIC) against *Staphylococcus aureus* ATCC 29213	−0.77	0.48	n.m.	n.m.
Minimum inhibitory concentration (MIC) against methicillin-susceptible (MSSA) *Staphylococcus aureus* MFBF 505	0.62	**−0.96***	n.m.	n.m.
Minimum inhibitory concentration (MIC) against methicillin-susceptible (MSSA) *Staphylococcus aureus* MFBF 10661	−0.06	−0.33	n.m.	n.m.
Minimum inhibitory concentration (MIC) against methicillin-susceptible (MSSA) *Staphylococcus aureus* MFBF 10666	−0.77	0.58	n.m.	n.m.
Minimum inhibitory concentration (MIC) against methicillin-resistant *Staphylococcus aureus* (MRSA) MFBF 101	0.32	−0.81	n.m.	n.m.
Minimum inhibitory concentration (MIC) against methicillin-resistant *Staphylococcus aureus* (MRSA) MFBF 124	−0.06	−0.33	n.m.	n.m.
Minimum inhibitory concentration (MIC) against methicillin-resistant *Staphylococcus aureus* (MRSA) MFBF 154	0.84	**−0.998****	n.m.	n.m.
Minimum inhibitory concentration (MIC) against methicillin-resistant *Staphylococcus aureus* (MRSA) MFBF 164	0.66	−0.91	n.m.	n.m.
Minimum inhibitory concentration (MIC) against methicillin-resistant *Staphylococcus aureus* (MRSA) MFBF 177	0.29	−0.66	n.m.	n.m.
Human MDA-MB-231 breast cancer cell line viability	**−0.81*****	**−0.72****	**−0.75*****	**−0.90*****
Human A1235 glioblastoma cell line viability	**−0.95*****	**−0.90*****	**−0.78*****	**−0.87*****

Significance: * $p < 0.05$; ** $p < 0.01$; *** $p < 0.001$; n.m. – not measured.

Table S3. Collection data for investigated samples of *G. alypum*, *G. punctata*, *G. cordifolia* and *G. meridionalis* from Croatia.

Collection Data	*G. alypum*	*G. punctata*	*G. cordifolia*	*G. meridionalis*
Sampling location	Konavle cliffs	Grobnik field	Baške Oštarije* or Alan, Velebit	Baške Oštarije, Velebit* or Grobnik field
Geographical latitude	42°30'50''N	45°22'39''N	44°31'41''N*, 44°43'15''N	44°31'41''N*, 45°22'39''N
Geographical longitude	18°19'07''E	14°30'53''E	15°08'38''E*, 14°58'05''E	15°08'38''E*, 14°30'53''E
Sampling period	March 2013	May 2012*, May 2013	June 2012*, May 2013	June 2012*, May 2013
Voucher No.	16 020	16 052*, 16 059_1	16 031*, 16 032	16 043*, 16 045_1

* Samples first collected and studied, used exclusively for anticancer potential evaluation.

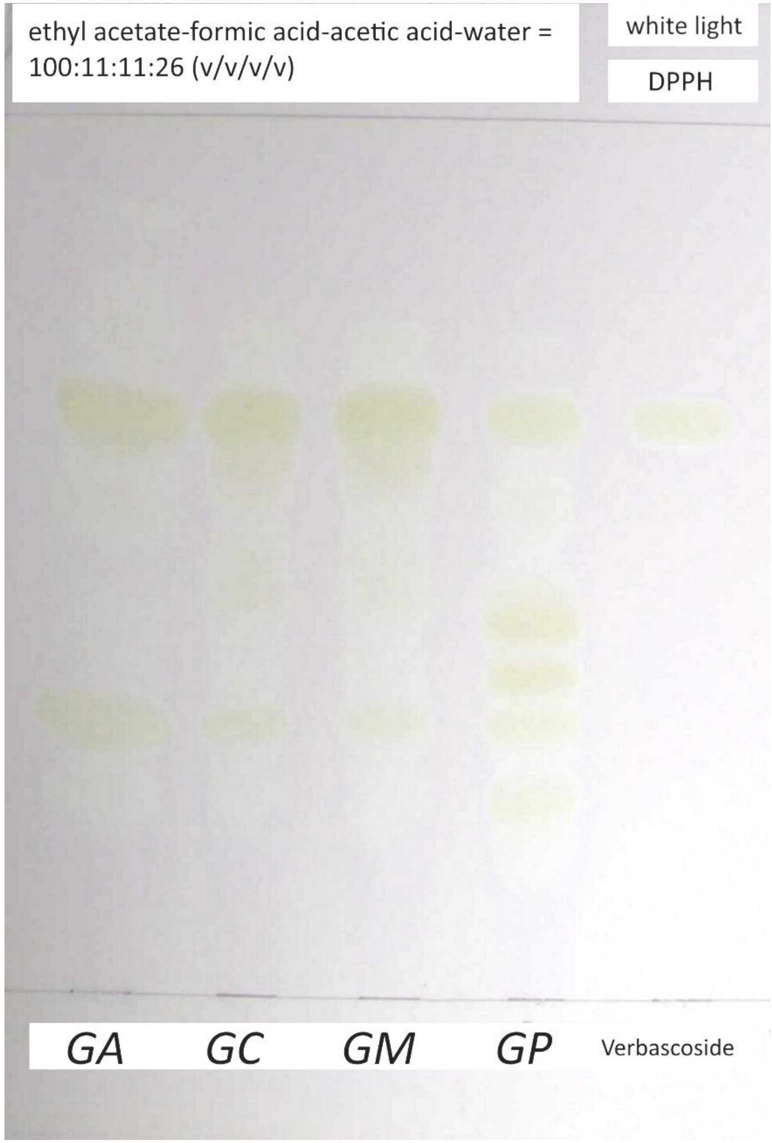

Figure S1. TLC chromatogram of Soxhlet extracts of aerial parts from *G. alypum* (*GA*), *G. cordifolia* (*GC*), *G. meridionalis* (*GM*) and *G. punctata* (*GP*) and verbascoside reference standard obtained after treatment with 0.05% (*w/v*) methanolic 2,2-diphenyl-1-picrylhydrazyl (DPPH), observed under white light. Stationary phase: silica gel 60 F_{254}; mobile phase: ethyl acetate-formic acid-glacial acetic acid-water = 100:11:11:26 (*v/v/v/v*).

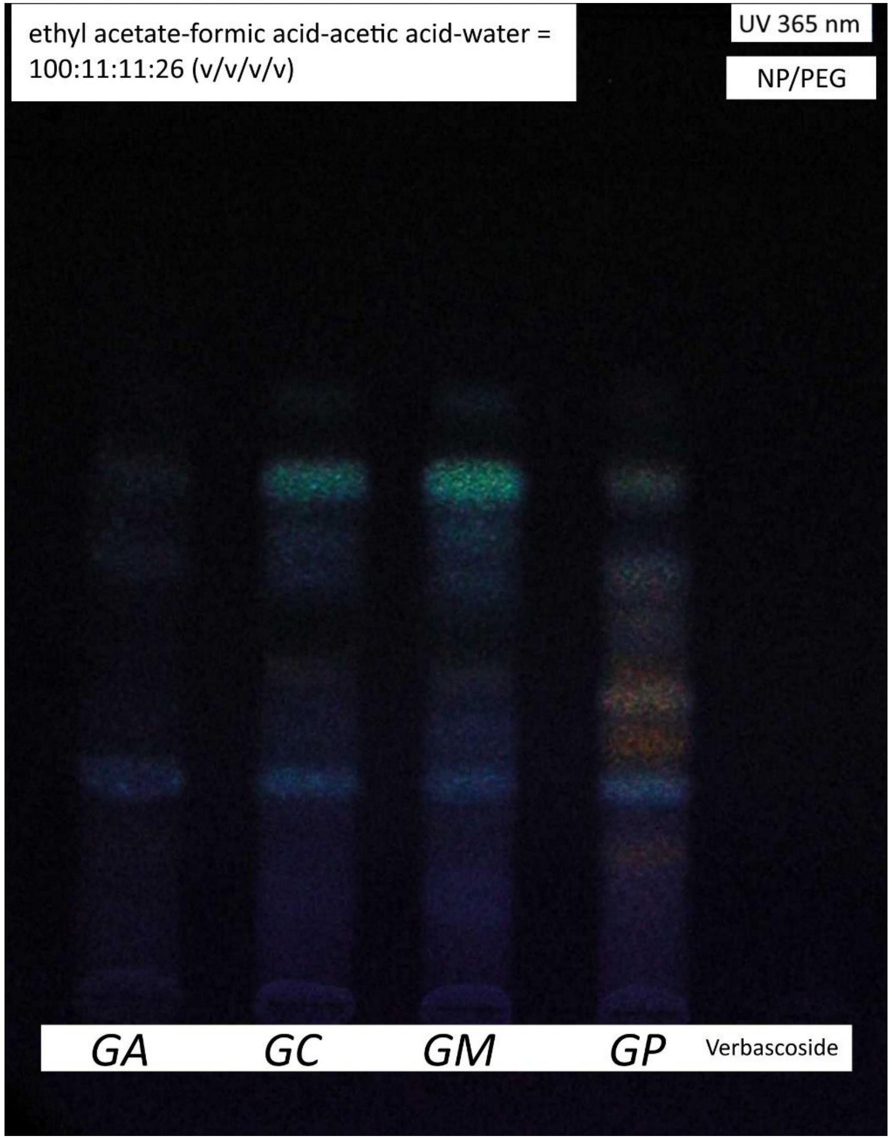

Figure S2. TLC chromatogram of Soxhlet extracts of aerial parts from *G. alypum* (*GA*), *G. cordifolia* (*GC*), *G. meridionalis* (*GM*) and *G. punctata* (*GP*) and verbascoside reference standard obtained after treatment with 1% (*w*/*v*) methanolic diphenylboric acid-β-ethylamino ester, and 5% (*w*/*v*) ethanolic polyethylene glycol 4000 (NP/PEG reagent), observed under UV 365 nm. Stationary phase: silica gel 60 F_{254}; mobile phase: ethyl acetate-formic acid-glacial acetic acid-water = 100:11:11:26 (*v*/*v*/*v*/*v*).
